# Oral Health Interventions to Improve Access in Rural Areas of High‐Income Countries: A Mixed Methods Systematic Review

**DOI:** 10.1111/cdoe.70058

**Published:** 2026-02-18

**Authors:** Amanda Kenny, Virginia Dickson‐Swift, Alexandra Carlin, David Nelson, Mark Gussy, Hewage Dona Vindya Gayathri, Sarah Baker

**Affiliations:** ^1^ La Trobe Rural Health School La Trobe University Bendigo Victoria Australia; ^2^ Institute of Health Lincoln Bishop University Lincoln UK; ^3^ Lincoln Institute for Rural and Coastal Health University of Lincoln Lincoln UK; ^4^ Institute of Oral Health, Ministry of Health Maharagama Sri Lanka

**Keywords:** access, oral health, rural, systematic review

## Abstract

**Objectives:**

The aim of this mixed methods systematic review was to identify oral health interventions in rural areas of high‐income countries and synthesise the evidence on how access is addressed.

**Methods:**

Searches were conducted in Cochrane, CINAHL, Dentistry and Oral Sciences Source, PsycINFO and PubMed, with the last search in January–February 2025. All study types published in English since 2000 were included that reported oral health interventions aimed at addressing access to dental services. The Mixed Methods Appraisal Tool was used to assess study quality. The Penchansky and Thomas model of access, with Saurman's adaptation, guided the thematic synthesis.

**Results:**

The final dataset was 73 articles. Most authors reported small‐scale interventions delivered by dental and primary health providers. Fluoride varnish application, treatments and health promotion were most reported in clinics, community settings and schools. Lack of service availability and accessibility caused by geographic distance required alternative service models, including telehealth. Free or minimal cost interventions were needed in low‐income settings. Stakeholder partnerships and understanding of local context were critical. Evaluations of community acceptability and awareness were rare. There was a dearth of studies addressing the six dimensions of access, with wide variation in study quality.

**Conclusions:**

There is an absence of robust, well evaluated studies, with lack of homogeneity preventing meta‐analysis. Rural oral health interventions should be informed by comprehensive frameworks of access, be grounded in equity, involve communities in design, development and evaluation, should reduce silos between oral and general healthcare, and should prioritise prevention. Access to high quality oral health is a fundamental human rights and equity issue for rural people.

## 1 Introduction

Universal access to high quality healthcare, including oral health, is a global, legal obligation [1]. Despite sustained calls to address a rising oral disease burden [2, 3, 4, 5], inequities affecting rural populations in high‐income countries remain underexamined [6, 7]. Rural people experience persistently poorer oral health than urban dwellers, representing an enduring equity and human rights issue [8, 9]. These disparities are driven by social determinants, including economic instability, inadequate education, poor healthcare access, lack of power and weak social supports [10]. In rural areas of high‐income countries, these factors are compounded by dental workforce shortages (reducing access and choice), high service costs, transport barriers, lack of water fluoridation, expensive healthy foods, limited access to oral health resources, and constrained primary health capacity [7, 11, 12, 13, 14].

While action is needed across all social determinants to reduce health inequities [15], access remains a key determinant requiring focused attention [16]. Penchansky and Thomas [17] conceptualised access as five interrelated dimensions: *Availability, Accessibility, Accommodation, Affordability and Acceptability*, reflecting the presence, reach, organisation, cost and cultural fit of services. Although widely applied, access remains a contested concept [18]. Critics highlight the emphasis on individual and service‐level factors, limited engagement with social determinants of health, and the interdependence of access dimensions. Strengthening one element does not ensure access [18, 19]. Despite these limitations, the framework [17] remains influential in examining interactions between users and services [18] and continues to inform oral health research [20, 21, 22]. Key adaptations of the framework emphasise users' ability to identify and engage with services through communication, information and health literacy, described as approachability [18] or awareness [23].

There has been no review of rural oral health interventions in high‐income countries through an access lens. This mixed methods systematic review addresses this gap by identifying rural oral health interventions and applying the Penchansky and Thomas framework [17] with Saurman's [23] addition of awareness, given the rural focus, to synthesis how access is addressed.

## 2 Methods

This mixed methods systematic review followed the steps of Khan, Kunz, Kleijnen and Antes [24]—framing questions, identifying studies, assessing quality, summarising evidence and interpreting findings. Given ongoing debates on how to define rural [25], in this review, we adopted a pragmatic approach, similar to other authors [26, 27], defining rural as any non‐urban location. Recognising significant differences in service delivery between low‐, middle‐ and high‐income countries [28], the review was limited to high income countries as classified by the Organisation for Economic Co‐operation and Development (OECD) [29] at the time of searching.

Reporting aligns with the PRISMA 2020 Statement for reporting systematic reviews [30] (see Appendix S1, PRISMA 2020 Checklist) and the review is registered with PROSPERO (CRD42022306594). PROSPERO was updated after registration to add Saurman's adaptation of the access model to the thematic synthesis.

### 2.1 Framing Questions

Using a Population/Intervention/Outcome framework to capture all interventions, rather than only those with comparators or controls, the *population* was rural people in high‐income countries. *Interventions* were defined as a programme, programme element or strategy designed to improve access to dental services, and *outcome* examined how access was addressed. Guided by Penchansky and Thomas's [17] definition of access, as the fit between clients and the system, inclusion criteria covered all study designs reporting on interventions targeting access dimensions—affordability, availability, accessibility, accommodation, acceptability and awareness. Reviews, opinion pieces, commentaries and grey literature were excluded, as the focus was on synthesising qualitative, quantitative and mixed method studies with transparent reporting.

### 2.2 Identifying Relevant Work

A librarian assisted in developing the search strategy. Preliminary searches in PubMed, and Dental and Oral Sciences Source refined keywords and Medical Subject Headings (MeSH) terms. Final searches were conducted in Cochrane, CINAHL, Dentistry and Oral Sciences Source, PsycINFO and PubMed, limited to English language studies with no date restrictions. Initial searches occurred in November–December 2022 and were updated in January–February 2025 (see Appendix S2, Search strategy for PubMed).

Search results were imported into Endnote and Covidence [31], where duplicates were removed. To improve reliability, one reviewer (AK) screened all titles, abstracts and full texts with other team members (VDS, AC, DN, MG, HDVG, SB) acting as second reviewers. Disagreements were resolved by consensus. Reference lists and citations were hand searched.

### 2.3 Assessing the Quality of Studies

Study quality was assessed using the Mixed Methods Appraisal Tool (MMAT) [32], a validated tool for assessing quantitative, qualitative and mixed method studies across core methodological criteria—clarity of the research question, appropriateness of the data to answer the question, and five specific criteria aligned to each research approach. Following MMAT [32] recommendations, no assessment scores were applied.

Although debate exists on quality assessment in mixed methods systematic reviews [33], we followed Thomas and Harden's [33] recommendation that while quality should be assessed, it should be acknowledged that there is a lack of evidence for excluding studies based on quality. Quality assessment was used to inform interpretations and synthesis, not to determine inclusion. This aligns with cautions [32, 33] that in mixed methods reviews, excluding studies due to methodological limitations can reduce richness and contextual breadth that is the hallmark of these types of reviews. We did, however, check for sensitivity in our synthesis [32, 33], ensuring that the themes reflected the data as a whole and were not heavily influenced by low quality study outliers. All team members independently assessed study quality, with a second reviewer confirming assessment, and conflicts were discussed by the team until consensus was reached.

### 2.4 Summarising the Evidence

Data extraction was completed by all authors (AK, VDS, AC, DN, MG, HDVG, SB) and included author, year, country, population and sample size, aim and/or research question, study design, site of intervention, intervention, results and key findings for each of the dimensions of access—*Availability, Accessibility, Accommodation, Affordability, Acceptability* [17] and *Awareness* [23].

### 2.5 Interpreting the Findings

Given the lack of homogeneity of the interventions and methods, a meta‐analysis of pooled data was not possible. Using the thematic synthesis approach of Thomas and Harden [33] PDFs of all articles were imported into QSR NVivo. Each article was coded line by line to capture the content and meaning of text and how it related to each of the domains of the Penchansky and Thomas [17] framework and Saurman [23] adaptation. In‐depth discussion occurred between two reviewers (AK, VDS) on the application of the codes. The codes were brought together and aligned with each of the dimensions of access, and after team discussion and agreement were written up as a thematic synthesis [33].

## 3 Results

**FIGURE 1:**
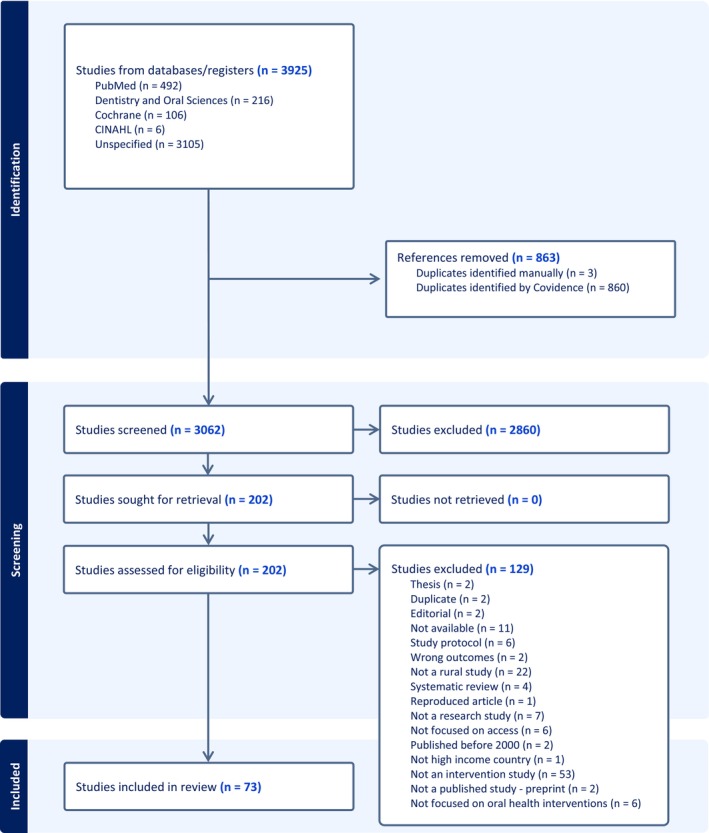
PRISMA Flow diagram showing search results.

The search results for the review are outlined in Figure 1.

A total of 73 articles were included, (see Appendix S3, Data extraction table). Across the studies, 58.9% (*n* = 43) were from the United States (US), 27.4% (*n* = 20) from Australia, with three from Canada, two each from New Zealand and the United Kingdom (UK), and one each from Chile, Korea and Taiwan. In Table 1, the major focus of the intervention, service providers, population and setting are summarised.

**TABLE 1:** Summary of interventions with citations.

Intervention	Service providers	Population	Setting
Application of fluoride varnish and sealant programmes [34, 35, 36, 37, 38, 39, 40, 41, 42, 43, 44, 45, 46, 47, 48, 49, 50]	Nurse practitioners and dietitians [35] Paediatricians [40] First Nations community workers or allied health professionals [36, 49] Dental staff [34, 37, 39, 41, 43, 44, 45, 46, 47, 48] Dental students [50] Multidisciplinary [38, 42]	Children [35, 37, 38, 39, 40, 44, 46, 47] First Nations children [34, 36, 37, 41, 42, 43, 45, 48, 49] Whole population [50]	Schools and/or family day care [34, 36, 37, 41, 42, 44, 45] Clinic [35, 38, 39, 40, 43, 46, 50] Multi‐site [42, 47, 48, 49]
Health promotion education for example, effective toothbrushing, appropriate food and beverage intake (avoidance of sugary drinks), appropriate use of bottles or sipper cups and importance of oral health check‐ups [51, 52, 53, 54, 55, 56, 57, 58, 59, 60, 61, 62, 63]	Volunteers [60, 61] Lay community health advisors [57] Allied health [53, 59] Dental staff [55, 62, 63] Aboriginal Community Controlled Health Organisations [58] Multidisciplinary [51, 54] Paediatrician [52] First Nations officers [56]	Rural farmers [62, 63] Intergenerational [59] Children [51, 55] First Nations children [52, 60] First Nations adults [56, 57] First Nations peoples [58, 61] Pregnant women [53, 54]	Clinics or health services [51, 52, 54, 56, 57, 63] Farming expo [62] Community settings [53, 58, 59, 61] Schools [60] Online [55]
Dental care via telehealth [64, 65, 66, 67, 68, 69, 70, 71]	Dental staff [64, 65, 66, 67, 68, 69, 70] Care home staff [71]	Older people [70, 71] Children [64, 65, 66, 68] Adults or unspecified [67, 69]	Aged care [70, 71] Clinic [64, 65, 66, 67, 69], Schools [68]
Treatment focused dental care [72, 73, 74, 75, 76, 77, 78, 79, 80, 81, 82, 83, 84, 85, 86, 87, 88, 89, 90, 91, 92, 93, 94, 95, 96, 97, 98, 99]	Dental staff [72, 74, 75, 77, 78, 79, 81, 82, 83, 84, 85, 88, 89, 90, 91, 92, 93, 95, 96, 97, 98, 99, 100] Dental students [86, 87, 94] Nurse led [73] Physician [76] Unclear [80]	Whole population [72, 73, 76, 77, 78, 79, 80, 84, 86, 94, 95] Adults [85] Children/adolescences [88, 89, 97, 98, 100] First Nations peoples [74, 83, 87, 90] First Nations children [42, 75, 81, 91, 96, 99] People with HIV [82, 93] Pregnant women [92]	Mobile clinic [72, 75, 79, 81] Onsite clinic [73, 74, 76, 77, 78, 83, 84, 85, 86, 87, 88, 89, 91, 92, 95, 96] Multi‐site [80, 82, 90, 93, 94, 99] Schools [97, 98, 100]
Medical dental integration [101, 102]	Multidisciplinary [101, 102]	Children [101] Adults [102]	Clinics [101, 102]
Education of non‐dental providers [103]	Aboriginal Health Workers [103]	First Nations peoples [103]	Multi‐site [103]
Remote assessments [104]	Multidisciplinary [104]	Children [104]	Clinics [104]
Client origin mapping [105]	Multidisciplinary [105]	Whole population [105]	Multi‐site [105]
Geofencing mobile technology [106].	Dental staff [106]	Whole population [106]	Clinics [106]

### 3.1 Appraisal of the Quality of Studies to Date

The earliest publication date of studies was 2002 [39] and the latest 2024 [55, 63, 66, 71, 104]. Full details of the quality appraisal for all study types are in Appendix S4, Quality appraisal of all study types.

#### 3.1.1 Quantitative Studies

The quality of the 55 quantitative studies varied widely (see Appendix S4, Quality appraisal of all study types). There were 15 articles (27.27%) where the MMAT screening questions were not met so no further quality assessment was made [46, 58, 59, 67, 68, 76, 78, 79, 87, 89, 94, 99, 100, 103, 105]. There were 11 (20%) quantitative studies where all MMAT criteria were met [40, 44, 48, 49, 50, 54, 61, 84, 88, 92, 95]. Sample sizes in the quantitative studies varied from 13 people [67] through to a study where 328 661 fluoride varnish services were reported [40].

#### 3.1.2 Qualitative Studies

There were ten qualitative studies spanning 2011 to 2023, with most meeting the MMAT criteria for qualitative studies, with the exception of Castillo, Echeto and Schentrup [73], where most data collected were quantitative outcome measures. Qualitative sample sizes varied from seven participants [63, 72] through to a study by Chi, Hopkins, Zahlis, Randall, Senturia, Orr, Mancl and Lenaker [74] with a sample of 141.

#### 3.1.3 Mixed Method Studies

Of the eight mixed method studies (see Appendix S4, Quality appraisal of all study types), none met all quality criteria. Most authors failed to address divergencies and inconsistencies between qualitative and qualitative results, the exception being Gaskin, Vazin, McCleary and Thorpe Jr. [80]. Where the sample size was given, they ranged from 48 children and clinic staff (no clear statement of number) [38] to 9750 children [98]. Gaskin, Vazin, McCleary and Thorpe Jr. [80] reported on a reduction of 18 562 inpatient stays following the mobilisation of community health workers, however, the total number of individuals was unclear.

### 3.2 How Access Is Addressed in Oral Health Interventions in Rural Areas of High‐Income Countries

The following synthesis uses the work of Penchansky and Thomas [17], with the inclusion of Saurman's [23] adaptation. The domains of access are not intended to be standalone constructs, so there is crossover content as each domain is interlinked.

### 3.3 Availability

Availability refers to the match between existing services and community needs [17]. A major barrier identified in the studies was the limited presence or capacity of dental services in rural areas [65, 66, 68, 74], prompting alternative service models. Examples included the Brighter Smiles program in Canada, where paediatric residents provided school‐based oral health education and fluoride varnish in remote First Nations communities [52]. In Taiwan, lay health advisors conducted oral screenings in underserved communities [57], while volunteer‐led programmes operated in regions like the Kimberley region in Western Australia [61]. While programmes were described, there was a dearth of studies that explored the match between services and need from the perspectives of the community.

A number of authors [38, 40, 43, 45] explored fluoride varnish application by non‐dental providers—nurses, nurse practitioners and Aboriginal health workers—improving preventative care for children [41, 43, 45], especially in non‐fluoridated communities [47]. It was argued that integrated medical—dental service models enhanced child oral health [101].

### 3.4 Accessibility

Accessibility refers to the physical and geographical factors affecting a person's ability to reach healthcare services [17]. Reported barriers included transport cost and availability [39, 69, 84], weather [36, 39, 104], culture [61, 81], communications issues [36], long travel distances [46, 65, 67, 84], time away from school/work and family [91] and unfamiliarity with larger towns [91]. In some Australian communities, travel distances reached 1000–1600 km round trips [46], severely limiting access to care.

Proposed solutions to strengthen accessibility included teledentistry [42, 65, 66, 67, 68, 69, 70, 71], outreach from dental therapists [74], mobile dental clinics [34, 46, 61, 72, 82], integrating oral health [38, 48, 51, 76] and co‐location of dental services with other primary health services [35, 46, 81, 85, 102, 105] to improve care coordination [80]. Notably, there appeared to be little community input into the development of solutions. Community‐based models [81, 96], the use of non‐traditional settings (e.g., schools, playgroups, community centres) for oral health screening and prevention activities [37, 38, 44, 45, 47, 72, 77, 90, 98, 99, 107], use of portable dental equipment transported in off‐road vehicles and trailers [34, 90] and community transport initiatives [95] reduced travel barriers and improved access.

### 3.5 Affordability

In Penchansky and Thomas's [17] framework, affordability refers to the cost of care and the ability to pay. Rural poverty and low‐income settings were major drivers for developing free or minimal cost interventions [36, 74, 76, 77, 81, 84, 86], with specialised services like orthodontics rarely accessible [64]. Many interventions were funded by philanthropy, government grants or insurance (e.g., Medicaid in the US) [63, 66, 74, 80, 81, 82, 100], with some relying on free volunteers to maintain service delivery [49, 60, 72, 90, 94]. Government advocacy to secure funding was critical [105] and maintaining services for high need populations a constant challenge [36]. Authors in the US [78], indicated that service viability was dependant on a mix of people who were self‐paying, government insured (through Medicaid) and uninsured.

Local care using dental therapists, portable equipment and local facilities was often most cost‐effective [34, 49, 70, 82]. In some studies, detail was provided on the capital cost of setting up an oral health intervention, for example, in Northern Ireland, a prototype teledentistry service was established at a cost of £25 000, however, no comparisons were made between the provision of teledentistry by a community dental service and an onsite service [69]. In the US [38], a fluoride varnish programme implemented by nurse practitioners and paediatric nurses cost US $3.56 per application compared to US$110–$240 for a restorative filling. The low cost of fluoride varnish application provided a reason to lobby the Australian Government for sustainable funding for school‐based fluoride varnish applications by dental assistants [45]. Preventative care improved oral health outcomes and were economically justified [39], particularly the cost effectiveness of fluoride varnish in rural areas without water fluoridation [47].

Incentives such as transport assistance and food increased participation in oral health interventions [36, 74, 84, 90, 99, 100]. In one US study [84], approximately US$31000 was expended annually on taxi services and prepaid fuel cards to improve service access. In some studies, children were prioritised for support such as transport [74, 94] with authors [44] arguing children were the highest priority when public funding was so limited.

### 3.6 Accommodation

Accommodation refers to aligning services to population need [17]. Key factors included ‘key player’ relationships [43, 52, 61, 92, 96], cross‐organisational partnerships [46, 55, 61, 82, 86, 98], supportive policies, adequate resource allocation, local strategic leaders, political will, interdisciplinary education [59, 105], the use of formal and informal channels [34, 52, 74, 84] and understanding of the local community [94]. While authors [82] argued that no single organisation could address access alone, particularly given the social determinants of health that impact poor oral health, the role of the community in developing services to meet their needs was not considered.

In school‐based programmes, teacher and parent engagement were critical [36, 37, 81, 100], with strategies including parent meetings, reminders and family liaison. Outreach workers and lay health advisors supported engagement and follow up [39, 48, 57, 65], particularly in Indigenous communities, respecting local culture and language [34, 41, 48, 52, 56, 61, 74, 81, 90, 96, 103]. In Australia, in‐depth knowledge of First Nations local norms was integral in accommodations, as reliance on conventional settings stifled community engagement [90]. Descriptions were given of face painting of children by dental therapists, with photographs of the children, with consent, displayed in the community to encourage participation [43]. In the US, Spanish‐speaking dental health coordinators working with community members in care coordination, case management and managing dental referrals highlighted the value of integrating community members in the service [101].

Resource roles supported busy clinicians engaged in direct clinical services [43]. Staff often undertook administrative and logistical tasks, including childcare [56], transporting children from school to scheduled appointments and follow‐up [37, 77], transportation assistance to support travel [34, 81], extensive work in referral tracking, follow up and retention [82], and flexibility around appointment structures [36, 90]. Adaptations by staff, included towing trailers with equipment for more than 40 000 km [34], and service delivery in unthreatening environments [78], including very small spaces [77].

Innovative models of accommodating the needs of communities included teledentistry (65% of older people avoided hospital‐based treatment) [69], the conduct of oral health assessments and fluoride provision by a paediatric nurse practitioner, a dietician and nursing and allied health students outside typical service delivery sites [35], and US farming community events to increase awareness of oral cancer [62].

### 3.7 Acceptability

Acceptability refers to the alignment between peoples' expectations of health services and what is delivered [17]. While providers were committed to improving access for disadvantaged populations, formal evaluations of acceptability by communities were rare, often relying on anecdotal provider perceptions [52]. When care was abandoned by clients, families' competing needs, the cost of transport, employment priorities and relocation were all cited as barriers [64].

Providing information and resources, such as toothpaste and toothbrushes, increased engagement and perceived acceptability [34, 36, 43, 54, 74]. High parent satisfaction was evident in US programs offering fluoride varnish, with requests to expand services to other family members [35]. Service demand for oral health promotion was viewed as a measure of acceptability [52]. Positive client experiences were influenced by provider manner and skills [74], and community connection [74, 96, 99, 101], including provider flexibility and passion for the community [101]. Accessing fluoride varnish in a paediatric practice [38] and teledentistry that reduced travel [65], contributed to client acceptability.

From the provider perspective, acceptability depended on reliable telecommunications connectivity, information technology support, especially when delivering teledentistry services [64] and sustainable staffing [105]. In the US, tailored interventions to meet the oral health needs of people with HIV/AIDS increased provider confidence and positive attitudes [82].

For First Nations populations, cultural safety, community consultation and partnerships with local workers were crucial for acceptability [34, 36, 37, 45, 48, 57, 61, 87, 90]. Engagement of Aboriginal education officers or lay health advisors enhanced consent, participation and client self‐efficacy [34, 37, 45, 57]. In the US, parental involvement improved over time through culturally responsive programmes, and this was viewed as a measure of acceptability [36].

### 3.8 Awareness

Saurman [23], proposed adding awareness to Penchansky and Thomas's access framework [17], emphasising that effective communication and information strategies, consideration of context and health literacy were key [23]. Authors of reviewed studies described diverse strategies aimed at awareness including the media and social media [34, 56], mobile technology and geofencing to distribute information [106], stakeholder presentations [59, 84], posters [62], home and community visits [41, 56], and direct contact with parents [42, 91, 100] with multiple follow ups [39]. Effectiveness of these strategies was rarely detailed.

Programme leaders often acted as advocates to encourage participation [77]. In many studies, recruitment relied on existing programmes [35, 38, 64, 75], with some programmes experiencing such high demand that no promotion was needed [78]. Language barriers [63] and poor phone, mail and internet access limited outreach, yet these mechanisms were used as core awareness strategies [34, 36, 73]. Key communicators—teachers, school principals and those who were Internet savvy [36, 37, 72, 77], public and primary health nurses and directors of nursing homes [72, 73, 77], and Indigenous or native speaking community members and elders [34, 37, 45, 52, 58, 63, 74, 81, 90]—were critical to awareness. Tailored, culturally relevant communication, including reminders in people's spoken language, was more important than written information [63]. Strategies like face painting, provision of resources to parents, and staff training raised community awareness [43].

Lack of awareness, by health departments and dental associations, of interventions occurring in their jurisdiction was viewed as a barrier to awareness [72]. Study modification, due to participants failing to attend treatment, was commonly reported [34, 64], with dental managers helping to address high no‐show rates, ensuring follow up and retention [82].

## 4 Discussion

The aim of this mixed methods systematic review was to summarise rural oral health interventions and how they addressed access. Most were small scale, heterogenous projects that limited the ability to draw definitive conclusions. Over half originated from the US, with only a few produced from other high‐income countries. Given that 43% of the population live rurally [108], the lack of rural oral health evidence is striking. Study quality varied considerably, underscoring the need for stronger, high‐quality research.

Despite decades of global strategies [2, 109, 110, 111], poor rural oral health remains a persistent problem. The WHO *Global Strategy and Action Plan on Oral Health 2023–2030* [111], aspires to equitable, affordable care, yet this review found implementation at community levels to be fragmented and poorly focused on access. The continuing inability of rural populations in high income countries to obtain dental care is indefensible [9, 112]. For example, 40% of rural adults had not accessed oral health care in over a year, and two‐thirds live in workforce shortage areas [112]. Most identified interventions were small, short term and insufficient to address widespread service gaps.

Using Penchansky and Thomas's [17] access framework, extended by Saurman [23], we found limited evidence that designers of rural oral health interventions consider all components of access—availability, accessibility, affordability, accommodation, acceptability and awareness—with little mention of the social determinants of health. Interventions only addressed single elements in isolation, weakening overall effectiveness. Affordability, accommodation and acceptability were especially weak links, as travel costs, limited transport options and lack of attention to what the community wanted were major barriers [17, 19, 113]. For some time, authors [114, 115] have documented access to transportation as one of the most critical factors in rural health care utilisation due to the costs of traversing geographic distances. Many studies depended on volunteers [72], reflecting chronic funding shortages.

Authors citing equity theory [116], suggest that poorer health outcomes should attract greater service investment; however, this review revealed the opposite. The disproportionate oral health burden carried by rural people [111], should mean wide rural access to oral health care. Few large scale or well evaluated national programmes were identified, and while prevention focused efforts were emerging, oral health promotion at population scale was scarce.

Although teledentistry is often promoted as a solution [112, 117], its potential is constrained by poor internet connectivity, lack of training and confidence and lack of reimbursement [118, 119]. The evidence reinforces that direct face‐to‐face early prevention and education remain essential in rural communities.

Some interventions successfully involved non‐dental providers and community health workers, improving outreach and accessibility [120]. However, integration between dental and broader health services was limited. Despite decades of global policy [121, 122, 123] on community involvement, the findings from this review are consistent with recent critiques [28], that community involvement in rural oral health remains tokenistic and underassessed.

### 4.1 Limitations and Future Directions

There was a lack of robust, well evaluated studies with consistent outcome measures, preventing meta‐analysis and limiting definitive conclusions. However, key themes were identified across a broad dataset. While using a recognised theoretical framework of access was a strength, the review revealed a gap between the framework [19, 23] and its application in rural contexts. The access dimensions and their interconnections were difficult to find, particularly links between affordability (service cost), accommodation (e.g., the cost of transportation) and acceptability. When interventions only focus on one link in the access chain [19] the likelihood of improved access falls. One link could be addressed (e.g., availability) while another is ignored (e.g., acceptability), leading to persistent poor outcomes. In future studies, the ‘weakest link’ principle should be explored and tested to see whether the overall success of an intervention in providing access is determined by its worst performing dimension.

Limited community engagement further highlights a potential theoretical gap, suggesting that access models [19, 23] may need to evolve to explicitly integrate equity and community co‐design as core, cross‐cutting principles rather than rarely acknowledged sequential elements.

This review focused on access, only one aspect of the social determinants of health. Future work should explore broader factors. Restricting inclusion to English language studies introduced language bias, and excluding grey literature may have limited insights, although such exclusions rarely alter review conclusions [124]. Including studies of varying quality enhanced contextual breadth, and while the synthesis was not heavily influenced by low‐quality study outliers, excluding lower quality articles might have produced different conclusions.

Funding applications should be prioritised that clearly demonstrate understanding of the social determinants of health, and the impact of these on all aspects of access. Funding responses must include evidence of community involvement in the design, delivery and evaluation of interventions.

## 5 Conclusion

This mixed methods systematic review identified a persistent gap between global oral health goals and rural implementation in high income countries. Despite well documented rural oral health inequities, evidence of interventions remains limited, fragmented and largely small scale. Most interventions were delivered by dental and primary care providers, commonly involving fluoride varnish, treatment and health promotion in community settings, clinics and schools, with limited community involvement. There was a dearth of studies that addressed all six dimensions of access, highlighting a clear theory‐practice gap. Future funding and interventions must be grounded in the social determinants of health, integrate all dimensions of access and meaningfully involve communities to achieve equitable rural oral health outcomes.

## Author Contributions

Amanda Kenny: conceptualisation, methodology, investigation, data curation, formal analysis, writing – original draft. Virginia Dickson‐Swift: conceptualisation, methodology, investigation, data curation, formal analysis, writing – original draft. Alexandra Carlin: conceptualisation, methodology, investigation, data curation, formal analysis, writing – review and editing. David Nelson: conceptualisation, methodology, investigation, data curation, formal analysis, writing – review and editing. Mark Gussy: conceptualisation, methodology, investigation, data curation, formal analysis, writing – review and editing. Hewage Dona Vindya Gayathri: conceptualisation, methodology, investigation, data curation, formal analysis, writing – review and editing. Sarah Baker: conceptualisation, methodology, investigation, data curation, formal analysis, writing – review and editing.

## Conflicts of Interest

Given their role as Editor on this journal, Professor Sarah Baker had no involvement in the peer‐review of this article and has no access to information regarding its peer‐review. Full responsibility for the editorial process for this article was delegated to an independent Editor.

## Supporting information


**Appendix S1:** PRISMA 2020 Checklist.

**Appendix S2:** Search strategy for PubMed.

**Appendix S3:** Data extraction.

**Appendix S4:** Quality appraisal of all study types.

## Data Availability

The data that supports the findings of this study are available in the Supporting Information of this article.
