# Supplementary material for: Oral Health Interventions to Improve Access in Rural Areas of High‐Income Countries: A Mixed Methods Systematic Review
Source: Community Dent Oral Epidemiol. 2026 Feb 18;54(3):273–84. doi: 10.1111/cdoe.70058 (PMC13146141; doi:10.1111/cdoe.70058)
Supplement: Supplementary file 2 — Appendix S2: Search strategy for PubMed. [file CDOE-54-273-s001.docx]

**Appendix 2 Search strategy for PubMed.**

| "Oral Health"[All Fields] OR "Oral Health"[MeSH Terms] OR "Dental Health Services"[MeSH Terms] OR "dental health service*"[All Fields] OR "Oral Hygiene"[All Fields] OR "Oral Hygiene"[MeSH Terms] OR "dental health"[All Fields] OR "dental care"[All Fields] OR "dental hygiene"[All Fields] OR "oral care"[All Fields] OR "mouth care"[All Fields] OR "dentist*"[All Fields] OR ("Dental Health Services"[MeSH Terms] OR ("dental"[All Fields] AND "health"[All Fields] AND "services"[All Fields]) OR "Dental Health Services"[All Fields] OR "dental"[All Fields] OR "dentally"[All Fields] OR "dentals"[All Fields]) OR "Dentists"[MeSH Terms] OR "Teledentistry"[All Fields]  AND  "Rural Health"[MeSH Terms] OR "Rural Health Services"[MeSH Terms] OR "rural*"[All Fields] OR "Rural Health"[All Fields] OR "rural health service*"[All Fields] OR "remote"[All Fields] OR "remotely"[All Fields] OR "remoteness"[All Fields] OR "remotes"[All Fields] OR "Rural Population"[MeSH Terms] OR "Rural Population"[All Fields] OR "rural communit*"[All Fields] OR "Medically Underserved Area"[MeSH Terms] OR "Non-urban"[All Fields] OR "nonurban"[All Fields] OR "nonmetropolitan"[All Fields] OR "Non-metropolitan"[All Fields] OR "geographic isolat*"[All Fields]  AND  "Health Services Accessibility"[MeSH Terms] OR "access to care"[All Fields] OR "access to health care"[All Fields] OR "access to healthcare"[All Fields] OR "access to services"[All Fields] OR "access to health services"[All Fields] OR "access*"[All Fields] |
| --- |
