# Supplementary material for: Oral Health Interventions to Improve Access in Rural Areas of High‐Income Countries: A Mixed Methods Systematic Review
Source: Community Dent Oral Epidemiol. 2026 Feb 18;54(3):273–84. doi: 10.1111/cdoe.70058 (PMC13146141; doi:10.1111/cdoe.70058)
Supplement: Supplementary file 3 — Appendix S3: Data extraction. [file CDOE-54-273-s003.docx]

**Appendix 3 – Data extraction**

| **Authors, year, country** | **Population Sample size**  **Intervention site** | **Aim and/or research question**  **Study design** | **Results** | **Affordability** | **Availability** | **Accessibility** | **Accommodation** | **Acceptability** | **Awareness** |
| --- | --- | --- | --- | --- | --- | --- | --- | --- | --- |
| Arrow et al., 2021 AUS. | Aboriginal communities (n=25) and children (n=388) involved in school dental programs and family day care. | To evaluate the feasibility, effectiveness, and community acceptance of a model of dental care based on atraumatic Restorative Treatment and the Hall Technique.  Parallel group, cluster randomised trial, stepped-wedge design with delayed treatment of control group. | 338 children participated (test 177, control 161) in 25 communities (test 12, control 13). Test children older, higher caries than control. 231 children examined at follow up. Follow up - children in test group had more filled teeth and decreased levels of decayed teeth. | No specific cost data but did consider economic issues. | Large need that does not match service availability. | Reports nontraditional site of service - library, daycare, mobile service to the community. | Important cultural factors reported. | Not measured but Aboriginal reference group guided the study. | Despite extensive community consultation and promotion of the study through social media, information dissemination, and snowball communication techniques, the study required modification due to failure to attend treatment/difficulty contacting participants. |
| Berndt, Leone, & King, 2008 USA. | 126 disadvantaged children. (30=general dentistry and teledentistry and 96 treated by orthodontic residents at  Washington State – Farm workers clinic, Children’s clinic. | To examine the feasibility of a general dental practitioner providing interceptive orthodontic services to disadvantaged children with real-time supervision from an orthodontist using teledentistry.  Case-control series study. | Both groups had significant improvements in PAR scores: 35.6% in the teledentistry group and 44.1% in the direct supervision group (P 0.001). There were no significant differences between the groups before treatment or after interceptive orthodontic treatment. | Both groups were Medicaid. Some had difficulty keeping appointments. | Teledentistry technical issues-unpredictable breakdowns, high degree of unreliability in connectivity. Need for onsite technical support. | Unsure, there were two sites (rural and urban) but many abandoned treatment with adequate progress because of their families’ competing needs for limited resources (e.g., moving, employment, transportation). | Interceptive orthodontic treatment supervised remotely through teledentistry was shown to be a promising approach. | Not evident | Participants recruited from existing program. Limited detail but noted that many people ceased to return for treatment. |
| Biordi, et al., 2015, USA. | 4360 children who were under 5, whose parents/guardians were enrolled in the Special Supplemental Nutrition Program for Women, Infants and Children (WIC) program. | To examine the feasibility of linking preventive oral health care services with services at two program sites; An interdisciplinary partnership that aims to improve access and provision of preventive oral health care for very young, poor and low-income children in Ohio.  Quality improvement project design. | Mean number of caries for the study population dropped between the first and second program visits and thereafter levelled off and remained relatively unchanged across the second and third visits. Reported daily tooth brushing or cleaning increased in both groups from visit one to visit three. | Medicaid. Budget cuts required a reduction in staff for some sites; hence, a redistribution of duties at any site would have to consider the associated cost---benefit ratio. | Approximately 225 children per year per site (mean=450 per year). Several parents inquired about bringing their older children to the site for similar examinations. | Yes, but staff were distributed between the sites to meet demand. | Need noted to older children and not provided (or within scope of study). | Not evident | Participants recruited from existing program. Limited detail. |
| Bradley, et al., 2010, UK. | 37/41 adults from Northern Ireland. Average age 79.6 yrs. Prototype teledentistry system to process oral medicine referrals, which was evaluated over six months. | To assess the feasibility of using teledentistry to process oral medicine referrals to a Belfast hospital consultant. Feasibility study-Service improvement. | Successful use of teledentistry, despite governance issues. Variable quality and quantity of patient information. Doctors provided better medical information than the dentists. Benefits to older people -increased domiciliary basis (negating expensive transport costs). | NHS: The capital costs associated with setting up the system were high. | Difficult to ascertain due pilot small sample. | Majority of people were assessed at teledentistry site, but if unable to travel domiciliary assessment were arranged. By providing locally based assessment, the need for expensive transport was avoided, accompanying staff time was significantly reduced and patient anxiety was alleviated. | There were inappropriate referrals such as one harmless lesion or referral to the wrong dental speciality. | Patient anxiety was alleviated. Potential to give a provisional diagnosis quickly, in familiar environments. | Not evident |
| Bright, & Lichtman 2024, USA | Service delivered to Latino farmworkers. Free community dental care network established by the Open-Door Clinic that reaches 100 people per year. Study conducted with 6 clinicians from the Open-Door Clinic and 1 co-ordinator at an advocacy organisation for Latine farmworkers. Rural catchment in Vermont, USA. | To explore the perceptions and experiences of clinicians about the impact of the COVID-19 pandemic on care delivery on a free dental care network established by the Open-Door Clinic.  Qualitative ethnographic study. | During the pandemic visits dropped by 50%. Some factors were pandemic related but there were structural and legal barriers. Lack of time off work, lack of transport, fear of immigration surveillance, language barriers. | Clinic was free. | Clinic only operated one day a week and was funded for only 6 people. Shift work and high numbers of hours worked by farm workers limited their access to the service. | Lack of transport was a major issue and fear that undocumented health workers would risk deportation. | Adapted health promotion to be culturally appropriate, created a Spanish language comic book to cover fundamental oral health knowledge/education, increased use of outreach visits to farms. Network has more than 140 volunteers -e.g., interpreters, van drivers, fundraisers. | Many workers had never seen a dentist prior to visiting the clinic. No specific detail on service acceptability as clinician perspectives only. | Language barriers impacted. Pamphlets were used for information sharing but it was noted that one on one interactions with trusted people was far more successful. |
| Bryant, et al., 2016, USA. | 518 parent-child dyads from the Navajo tribal members from a native American reservation in the USA. | To evaluate the intervention using up to 3 years of follow up data.  Mixed methods, cluster randomised controlled trial. | Child participation was good FV:79.7 (cohort 1) and 85.3% (cohort 2) received at least three of four applications; OHP 74.5 (cohort 1) and 78.4% (cohort 2) attended at least three of five events. Parent participation was low 10.5 (cohort 1) and 29.8% (cohort 2) attended at least three of four events. Qualitative analysis from native staff reported barriers (e.g. geographical expanse, constraints of a research trial) and suggested strategies to improve parent participation. | Not evident | Contacting parents remained a constant issue. Greater incentives could have been used, such as incentives to participate - having food available at parent events or assisting with transportation. | Difficulties in maintaining a high level of parent attendance in classroom activities, few other options exist regarding accessible and convenient locations on the reservation for engaging both parents and children. However, the Community Oral Health Specialists made parent events more accessible and convenient by working with staff to schedule events to coincide with other community events. | The researchers speculate that parents were less likely to attend intervention events than their children because parents had more schedule conflicts, couldn’t take time from their day’s responsibilities, and needed to find their own transportation. | Characteristics of individual parents influenced participation in the intervention (e.g., improved parent participation associated with smaller household size etc. | Strategies to communicate with parents included reminder flyers sent home with children, personal invitations, continued reminder calls and texting, however poor phone reception, disconnected phones, changing numbers was a major challenge. Teachers were critical in information sharing. |
| Carr, et al., 2008, USA. | Staff from 33 mobile dental programs (21 program directors). | To identify and describe mobile dental programs in California.  Cross-sectional survey | 33 programs identified, with a survey response rate of 70%. The populations most likely to be served were those with low-income (100%); elementary (77%); preschool (68%); children and English speakers (64%); Medicaid eligible (64%). | Medicaid-eligible (64%). Study recognises that the lack of reporting on financial data was problematic. More cost and revenue data are needed to determine how many programs are profitable and sustainable and what type of subsidies are provided | Mobile dental clinics were currently serving at least 30 of the 58 counties in CA. At least 50% of the program directors reported serving areas designated as dental health professional shortage areas. | Dental clinic clinics predominantly held in schools (91%). 65% indicated that their program had increased access to care for their target population by a large extent; 26% a moderate extent and 9% not at all. | Most program directors indicates that if their program was discontinued, it would be "very difficult" (61%) or "difficult" (35%) for their target populations to get dental services. Significantly, the best way to identify the dental programs was by using the internet, but dentally underserved populations with low income and low health literacy may be less likely to find out about these services | Not evident | Public health nurses were critical for promotion about the service. The best way to promote services was the Internet but many who have low incomes, low health literacy less likely to find out about services. Dental societies and health departments weren’t able to provide information although in their jurisdiction. |
| Castillo et.al., 2023, USA | 118 people receiving a dental referral to the student led clinic. A nurse led practice in partnership with the University of Florida College of Dentistry. | To evaluate the partnership impact and barriers to access.  Qualitative design. | 118 referrals were made. 45 (38%) people completed their appointment. Common barriers to completing an appointment were cost (32%) and time constraints (24%). | Dental care is provided at a reduced fee to those who are uninsured and those with low incomes. | Not evident. | Not evident. | Developed a primary care workflow to streamline access. | Limited detail on acceptability. | Recruited from a general clinic with nurses asking whether they had accessed dental care. Contacted by telephone, patient portal or by mail. |
| Chen, & Fricton, 2007, USA. | 13-unknown demographic. 24 teledentistry clinic visits. University of Minnesota School of Dentistry | To create a teledentistry network linking University of Minnesota School of Dentistry specialists to dental practitioners and patients in sites in remote rural areas where access is to care is problematic.  Pilot project, survey. | The overall patient satisfaction that teledentistry met their current healthcare needs was rated 6.9 on average (1=not satisfied at all, 7=completely satisfied). | High levels of satisfaction with less cost due to reduced travel hypothesized. | Average distance for people to the teledentistry clinic was 13 miles. Significant reduction from 200-300 one way to university. | Addresses rural travel issues. | High levels of satisfaction. Approximately half of the people reported that they would not have preferred to see the specialist in person mainly because of no perceived necessity and that teledentistry provided similar 'in-person' experience. | Not evident. | Not evident. |
| Chi, et al., 2019., USA | 16 health providers with experience providing care in Alaska's Yuton-Kuskokwim Delta (YK Delta) and 125 community members from six YK Delta Communities.  . | To evaluate perceptions of the dental therapy programme in Alaska's YK Delta by interviewing health providers and community members  Evaluation design. | Providers believed individuals have benefited from clinic‐based restorative care and community‐ based education provided by dental therapists. The restricted scope of dental therapy practice limits the complexity of care that may be offered. However, community members expressed high satisfaction with the quality of care provided by dental therapists. | Most individuals in the YK Delta are eligible for Medicaid. | Increased provision of hygiene supplied. | Interviewees from all communities believed dental care access for children in the YK Delta is good because air travel to Bethel is covered by Medicaid. Although care for adults is restricted to emergency treatments. | The YKHC dental care system requires the hiring of additional dental therapists as well as dentists to be optimally staffed and to meet the dental care needs of community members. | Almost all participating community members self‐identified as Yup'ik (98.4%) and 1.9% identified as Cup'ik. In terms of language preference, 30.4% preferred Yup'ik, 29.6% preferred English, and 40% had equal preference for Yup'ik and English. Women represented 68.8% | Limited information but having someone that can speak the local language helps |
| Croucher& Ackermann 2006, NZ. | 15 locations, consisting of Northland schoolchildren (predominantly Maori). | To investigate the feasibility and benefits of engaging a mobile community dentist to accept referrals of children who required treatment beyond the scope of practice of dental therapists.  Evaluation design. | The program ran for 71 days, of which 49 were clinic and 22 were administrative. Dental therapists and assistants saw the programme as a success because they had a reliable referral pathway for ongoing care of their patients. All feedback received from parents and patients were favourable. Ability to deliver care on school sites was seen as the programmes biggest advantage. | Financial viability was not the principal criterion for the success of the program but noted the importance of low fees. | The model of service delivery used proved to be successful in providing equitable access to advice and treatment from a dentist for children experiencing some of the highest dental needs in Northland. | Mobile dentist drove two-three hours a day but was able to reach 15 locations some of which were very isolated rural schools. | The income derived from fees claimed under the various contracts was less than costs of the program. | Favourable views. | Referral program via dental therapists. Parents notified with the referral. Limited detail provided. |
| Davis, et al., 2010, USA. | 1655 patients from a rural eastern Oregon, USA - Private Family Medical Practice were included in the study. Clinic providers conducted a basic oral health screening and patients, or their caregivers, completed a self-report survey about dental needs and access to care. oral health conditions. | To describe the prevalence of oral health conditions and unmet dental needs among patients presenting for routine care in a rural Oregon family medicine practice.  A cross-sectional design (screening/observation and self-report survey). | Half of the patients who were screened (46.0%, n = 310) had oral health conditions detected, including partial edentulism (24.5%), dental caries (12.9%), complete edentulism (9.9%), and cracked teeth (8.9%). 28% reported experiencing unmet dental needs. Patients with dental insurance were significantly more likely to report better oral and general health outcomes as compared with those who had no insurance or health insurance only. | No data on wider implications for affordability of dental care. Although it should be noted that there were no additional costs associated with study participation on this occasion. | Oral health conditions are common in people who present for regularly scheduled visits in rural primary care. Half of the people who were screened had oral health problems and 28% reported unmet dental needs. | Primary care settings may present opportune environments for reaching people who are unable to obtain regular dental care. | The service was more about identifying needs (28% reported unmet dental needs) as opposed to accommodating those needs. | Not evident | Recruited from a private family medicine practice. Limited detail on awareness. |
| Delinger, et al., 2014, USA. | Total sample, n=8. Kansas state. | To explore the experiences of the Extended Care Permit (ECP) dental hygienist in the state of Kansas.  Qualitative design. | Seven major categories emerged from the data analysis: entrepreneur dental hygienist, partnerships, funding, barriers, sustainability, models of care and the impact of the ECP. The findings of this study revealed that ECP hygienists are making an impact with underserved populations, primarily children, the elderly and special needs patients. | No data on whether this supported client’s ability to pay but the ECP providers reported their own financial reimbursement as a barrier. | The ECP provider is working with targeted populations who have limited or no access to dental care. | The ECP dental hygienists increased access to preventive oral health services to those in unserved and underserved areas of Kansas. | The findings indicated that ECP hygienists are making an impact with underserved populations, specifically with (1) children (2) older adults (3) people with special needs | Limited detail about patient acceptability. Provider perspectives were mostly positive with regard to the impact they are making with underserved populations. | Described leaders as trailblazers and entrepreneurs whose role it was to convince people to participate but little further detail provided of strategies used. |
| Dimitropou et al., 2019, AUS. | 104 Aboriginal children aged 5-17 who attended three schools in Central Northern NSW, Australia participated in a school-based fluoride varnish program. | To determine if a school-based fluoride varnish program can provide Aboriginal children with at least three fluoride varnish applications over 12 months and whether the routine application of fluoride varnish for Aboriginal children in schools is a feasible approach for oral health promotion in Aboriginal communities. Quantitative design - Repeated non-randomized intervention (routine application of fluoride varnish in a school setting). | Study took place in 3 schools from Jan-Dec 2017. Consent response was 100% in schools where an Aboriginal Education officer was enlisted to liaise with families to seek consent to participate. 52 of the children were male and 52 were female, majority aged 5-12 (76%). Majority of children (65.4%) received at least three fluoride varnish applications. On average 70% of students received an application of fluoride varnish on each "fluoride varnish day". | No direct cost to the client but Aboriginal children face limited access to dental services, high costs of toothbrushes and toothpaste in rural/remote areas. Not clear on the cost of the program itself. | Aboriginal Education Officers played a key role in increasing student participation and could be useful in other oral health promotion programs. | Delivery in the school-based setting enabled a systematic approach which increased the likelihood of children receiving 3 or more applications of fluoride varnish. Aboriginal Education Officers increased student participation in the schools where they were used. | Could be a feasible approach to reducing dental caries and improving the oral health of Aboriginal children living in rural/remote Australia. Aboriginal children in NSW have over 3 times the amount of dental caries of non-Aboriginal children in NSW. | Not evident | School principal managed the consent process with an Aboriginal Education Officer engaged to liaise with parents. No detail of strategies used just they were crucial to program success. |
| Fallon, et al., 2010, USA. | Residents of 6 rural county health districts in Northwest Ohio, USA. Between 2004-2007 the clinic had 10,487 patient visits. Clinic offers most basic dental procedures, most common are restorations (fillings), examinations, cleanings and tooth extractions. | To describe how a full-service dental clinic was established to serve six rural county health districts in Northwest Ohio, USA. Quantitative descriptive study on implementation and utilisation of a dental clinic. | Retired volunteer dentists are instrumental in the success of creating the clinic, staring with a field type operation in 2001. On an average day each dentists treats 12-15 people, each clinic hygienist sees 13-16 people. 71.3% of clinic participants rely on Medicaid but cannot receive services because of the small number of dental practices that treat Medicaid. Clinic fees have increased each year using the govt's consumer price index. | A sliding fee scale that is based on a percentage of the federal poverty rate is used. People at the top of the sliding fees scale pay the same fee as those being treated by private dentists. To ensure financial stability, the clinic strives to maintain a ratio of 70% patients with Medicaid to 30% of those who pay according to the sliding fee schedule. | Even with no clinic promotion or advertising, a large volume of patients is in need of services. The large number of patient visits 10,487 over 2004-2007 is evidence. | 10,487 patients seen 2004-2007 who would have been unlikely to have access to dental services. | Recruiting and retaining dentists is the most challenging issue for the clinic and dentists can’t always be maximally productive due to space limitations. | Not explicitly reported but important to note about support from the local dental community and the importance that private practices do not see this as a competitor and have embraced it which is evidence of wider acceptability from dental community. | Stated no clinic promotion or advertising. Relied on referrals only |
| Gardner et al., 2012, USA. | In 2009, 2900 patients were seen (3165 patient encounters) in addition to 268 telemedicine consultations. The Health Wagon provides.  Mountains of Southwest Virginia in Central Appalachia. | To describe the access to care issues of the population living in Southwest Virginia in Central Appalachia and the innovative approach of the Health Wagon designed to address the problem of access to health care. Quantitative descriptive study | In 2009, it provided over $2.2 million in free care. In relation to dental care this included 1545 episodes of general dental care, 4893 extractions, 2113 fillings, 278 teeth cleaning, 1545 dental examinations only, 35 dentures. | Primary healthcare services, prevention, and education are provided free of charge. 61% reason for attending the Health Wagon was that they had no insurance and could not afford health care. Funding has been challenging. | Available staff and resources are small compared with the need/demand for services. | The mobile clinic is the primary means of the Health Wagon health care delivery. It visits 8 sites across Southwest Virginia. Health fairs are also held at community centres and churches close to where people live and work. | Given the rural area it is difficult for the organisation to attract staff with specialised skills (e.g., healthcare professionals, ICT support and grant writers). Difficulties obtaining electronic medical records in the mobile clinic. | Not evident. | Services provided at health fairs to raise awareness. |
| Gaskin, et. al., 2018, USA. | Residents of five designated Health Enterprise Zones (for underserved groups) in the State of Maryland, USA. | To examine whether the Maryland Health Enterprise Zone Initiative was associated with reductions in hospital use.  Mixed methods including quantitative quasi-experimental design using hospital inpatient stay data and emergency department visit data , Qualitative component using structured interviews and focus groups. | The initiative was associated with a reduction of 18,562 inpatient stays and an increase of 40,488 emergency department visits in the period 2013–16. | There was a net cost saving from reduced inpatient stays which far outweighed the initiative's cost to the state. The state used funds to benefit the community via incentives to attract healthcare providers to underserved communities. Likely reduced the use of costly inpatient care. | Limited access/availability before. The program supported the coordination of health care and social services for vulnerable populations. Although the initiative does not report in great depth the dental services that were offered. | The program was associated with improved access to care and reduced inpatient admissions and their associated costs. | Residents started becoming aware of their health, exercising more, and monitoring their diets. Providers also felt that the initiative helped patients manage chronic conditions. | The qualitative findings stated that residents and health care providers indicated that the initiative improved access to care and enabled residents to adopt health behaviours and practices that improved their health outcomes. | Not evident. |
| Geiger et al., 2019, USA. | Analytic extract of preventive Medicaid claims for 6,275,456 children younger than 6 years in 39 US states. | Using a multistate sample of Medicaid claims for young children eligible to receive preventative oral health services in medical offices to examine receipt of preventative oral health services in medical offices and dental offices by county rurality. Additionally, to evaluate a child's odds of receiving preventative oral health services in a medical office by county rurality, while controlling for other patient and county characteristics.  Quantitative cross-sectional design. | Preventative oral health services in medical offices were received by 7.8% of children. Rates of preventative oral health services in medical offices were higher in metropolitan (metro) counties (8.4%) than nonmetro adjacent to metro (5.8%) and non-metro not adjacent to metro (4.3%). In adjusted analysis, children living in nonmetro not adjacent to metro (OR = 0.79, 95% CI: 0.64-0.99) and adjacent to metro counties (OR = 0.70, 95% CI: 0.59-0.82) were significantly less likely to receive preventative oral health services in medical offices than children living in metro counties. | Children living in counties with a high degree of poverty were less likely to receive preventative oral health services in medical offices than residents of other counties. | Preventative oral health services were found to be lowest in nonmetro counties. Children living in nonmetro counties were less likely to receive preventative oral health services in medical offices than children living in metro counties. | For a small number, some Medicaid enrolled children are receiving preventative oral health services in rural areas. The rate was lowest for those in the most remote counties (4.3%). | Not evident | Not evident | Not evident. |
| Gnaedinger,2018, USA. | Patients at a paediatric practice in rural Vermont, USA. The sample comprised patients aged 9,18,24 and 30 months who were seen for their well child visit and staff at the practice. varnish application. | To demonstrate cost-effective implementation of a fluoride varnish application program at a medium-sized, rural paediatric practice. Quantitative (observational) and qualitative study design. | 56% (n=27) of sample received fluoride varnish at their visit. Qualitative themes included the following: reasons subjects did/did not receive fluoride varnish, ease of fluoride varnish application, increased oral hygiene education, visit flow, application time, older children's fluoride varnish need and billing issues. | The total project cost was $230.98, and the cost was borne by the paediatric practice. 43% of patients at the practice were part of Vermont's State Children's Health Insurance Program. | Majority of paediatric practices do not routinely apply fluoride varnish at well child visit and young children are not receiving preventative dental care especially in rural and poor areas. This program in rural Vermont is in an area where children normally have inadequate access to dental care. | Children do not normally have access to adequate dental care in places such as rural Vermont. This has improved access. | Using nurse practitioners and nurses in paediatric practices where children have inadequate dental care is feasible and economical, and more than half of the children in the study received fluoride varnish. | There was a positive reception by parents. In the post project focus group, the clinic staff unanimously voted to continue the fluoride varnish program. | Not evident. |
| Harrison, et al., 2006, Canada. | Children from a remote First Nations community in British Columbia, Canada - Hartley Bay (Gitga'at), part of the Tsimshan Nation. | To improve children's dental health in a remote First Nations community in the context of a service-learning experience for Paediatrics residents.  Quantitative descriptive/observational study design. | Only 31% (4/13) of prekindergarten and 8% (2/26) of kindergarten to Grade 12 children had no dental caries. Planning of the Brighter Smiles intervention involved community leaders, teachers, parents, Elders, health care staff, paediatrics residents, and dental and medical faculty from the University of British Columbia (UBC). Program includes school-based brush-ins, fluoride programs, classroom presentations, and regular visits by paediatrics residents to provide well-child care that includes age-appropriate dental counselling to parents at the clinic visits. | No data on the price of service/cost to clients, it was funded from a project grant. Not clear if there were any direct costs to the community itself. | Children's dental health identified as a concern/need by the Hartley Bay community (Approx 200 people) and early indicators show that the service in its current form has improved children's oral health. Consultant dental hygienist has informally reported noticeable improvements in oral hygiene in the majority of children from the community. | Prior to Brighter Smiles, regular paediatric services were not available in the community. Transportation in and out of the village is by boat or float plane. | The service was a direct response to a need expressed by the First Nations Community of Hartley Bay. Community members identified children's dental health as an issue of concern. | Not reported explicitly but a follow up dental health survey is planned as part of program evaluation after Brighter Smiles has been in place for a few more years. Although it should be noted that Brighter Smiles has contributed to community capacity building. | Limited information but heavy reliance on Elders within the community for program planning. |
| Irving, et al., 2017, AUS. | A total of 49 Aboriginal children aged 4–14 years attending the new collaborative oral health service in communities of Northern Central Tablelands of NSW during October to December 2014. | To examine the views of children (and parents) who accessed the service, including: the extent of reported dental problems, oral health knowledge, attitudes and behaviour, accessibility of oral health services, satisfaction and cultural sensitivity of the service.  A survey of the children who accessed this service | A total of 49 (71%) Aboriginal children aged 4–14 (or parents of), provided responses to the survey. All agreed that healthy teeth were important (100%), but many thought oral disease leading to extraction was normal (68%). High levels of oral pain were reported (66%), half (53%) reported brushing morning and night. Access to the new dental health service was reported as ‘easy’ (92%). Many walked (47%) or were driven (35%) in <30 min (90%). | Not evident | Not evident | Almost all participants reported that it was easy to access the new dental health service | Culturally appropriate design. | All 49 respondents reported that they were happy with their treatment and felt that they had been treated in a culturally competent manner with their Aboriginality respected. | Heavy reliance on community Elders in program design and delivery |
| Javed, et al., 2024, UK. | 352 triage forms from children under 16 undergoing general anaesthetics for extractions in rural UK. | To evaluate the orthodontic triage assessment form and complete a service evaluation on the impact of the form.  Mixed method study. | Of the 352 forms analysed, 90.6% of children did not require face to face assessment. 9.4% were triaged to attend face to face and 78.8% had their treatment plan modified. | Reduced costs associated with travel | Failure to recruit dentists has led to service pressure but by carrying out remote assessments staff can identify who needs face to face consultations. | Remote assessment reduces travel and other time for rural people. | Not evident | Staff indicate that patients value the low incidence of travel. | Limited detail as service operates via referral. |
| Jones, et al., 2012, USA. | 473 people with HIV/AIDS, 153 dental hygiene students participating in the Innovations in Oral Health Care Initiative in rural Oregon, USA. | To increase the number of people with HIV seen and the number of dental providers trained. No clear study design. | A total of 473 patients (65% of their possible target population). 1800 hours of direct clinical care was provided. | Leveraged multiple funding sources to fund – ongoing source of revenue. Program staff helped people buy bus tickets etc to attend and gas cards and hotel rooms were provided for those who had to travel long distances. | Incorporation of dental hygiene. | A dental case manager was employed to help people access the service. People were shuttled to the service from remote locations; clinicians were sent to satellite clinics in the second year to reduce transportation need. | Designed as a partnership between a HIV Alliance, community health centres with existing dental services and a community college. Formal partnership agreement with clear documentation of roles. | Patient satisfaction surveys were conducted – they reported that students were professional, took their time and provided full explanations. People with HIV noted a lack of discrimination. Pre-post surveys were conducted with students – less stigma and increased understanding of lack of dental care access. | Following high number of no shows, dental case manager position expanded to follow up no shows and retail those using the service. Served as a link between the dentist and clients. |
| Ju, et al., 2017, AUS | 400 Indigenous adults – 203 intervention, 197 control. Regional location in South Australia. | To determine the effect of an oral health literacy intervention on oral health literacy outcomes among rural-dwelling Indigenous Australians. Randomised controlled trial. The primary outcome was oral health literacy as assessed by the HeLD-14 instrument.  Self-reported questionnaires at baseline and 12 months. | 293 (73.3% completed the 12 month follow up – 74.4% intervention group, 72.1% control. Hypothesis that a context specific health literacy intervention would improve oral health literacy proved only partly true. | People received a $10 gift voucher for each intervention session they attended. | Intervention was community based. | Range of strategies used for recruitment - word-of-mouth, key stakeholder presentations, home visits, visits to community centres, posters in community centres, advertisement on local radio station and self-nomination. | Not evident | Not evident. | A range of strategies used to raise awareness including word of mouth, key stakeholder and community centre presentations, home visits, use of the local media, including radio, and posters in community centres. Limited detail on success of these strategies. |
| Kaakko, et al., 2002, USA | 438 parents consented for their children to participate in the Access to Baby and Child Dentistry Program in rural Stevens County, Washington, USA. | To assess utilisation of program, average dental expenditure per child, and oral health status. Randomised trial – utilisation and expenditure data extracted from Medicaid system. After two years in the program parents were contacted for their children to be clinically assessed. Randomised trial. | A 38% higher level of utilisation occurred in program children compared to other Medicaid children. Program children had significantly more claims for fluoride varnish than children not in program. Little cost difference between children in program and other Medicaid enrolled children. 276 clinical examinations were conducted after two years of early intervention – children in program had significantly fewer teeth with initial caries than non-program children. | Many parents were not aware of the benefits that their children were entitled to. | Many parents were not aware of the services. | Transportation is a major barrier to attendance. Distance from major towns and impact of harsh winters. | Preventative treatment increased in first period but % of children who received fluoride treatment was below program goals. Staff had difficulty in adapting to a higher level of visits for preventative service. | Oral health prevention is not well established with low-income families who only respond to emergency situations. | Children identified through social and health services. Parents written to, with written follow ups and then by telephone. Up to 20 attempts were made to reach parents. Noted that their outcomes were impacted as many parents are not aware of entitlements to dental care. |
| Kranz, et al., 2022, USA | Sample of children aged 1-5 328,661 visits. Rural counties in USA. | To examine variation in the delivery of fluoride varnish during medical visits. Observational study using private insurance claims for children aged 1-5. Logistic regression used to estimate the odds of a well child visit including fluoride varnish by rural county, with adjustments for individual and country characteristics. | Fluoride varnish was included in 6.2% of visits in rural areas and 10.3% in remote. Well child visits in rural areas were more likely to include fluoride varnish than children in urban areas. Results are promising as rates of annual visits for children 1-5 are lower in rural areas compared to urban. | Not evident. | Included as part of well child visits. | Insurance coverage might not include preventative services. | Included as part of well child visits. | Not evident. | Not evident. |
| Kruger, et al., 2010, AUS | 1405 people seen from 1999 to 2004. Comparing service provision in Australian Aboriginal Medical Service and community clinic. Rural and remote Western Australia. | To compare oral health services between the Aboriginal Medical Service clinics and typical rural community clinics. No clear method stated. Analysis of patient demographic and treatment data. The values of each service item were calculated based on the Department of Veteran Affairs fee schedule for dental services. | Four out of five clinics were Australian Aboriginal Medical Services. The clinics treated a higher proportion of emergencies than community clinics. 5867 treatments were provided in the Australian Aboriginal Medical Services. The value per treatment was higher in community clinics. | The average values of treatment ranged from 0-$929. | 5867 treatments were provided in the Australian Aboriginal Medical Services. | Not evident. | The majority (95.3%) of people receiving services in the Aboriginal Medical Services were Aboriginal peoples. | Not evident. | Not evident. |
| Lalloo, et al., 2021, AUS. | 408 Indigenous children. 196 consented to the epidemiological examination and intervention (intervention group), 212 in in the comparison group (epidemiological examination).  Far North Queensland Australia. | To assess the effectiveness of an annual intervention on caries increment at 2 year follow up. Non-randomised preventive trial | 51 % of children retained at 2 years (60% intervention, 43% comparison). Children in the intervention had fewer new surfaces with caries lesions although not statistically significant. | Nil cost to children | Active treatments provided. | Children were examined in school classrooms. | The team visited the community for 3 weeks each year after the wet season. | Not evident. | Stated that there was extensive consultation with the community but no further detail. All school children in the community were invited to participate via detailed information sheet sent to parents. Noted that recruitment is often impacted by inability to contact carers/disinterest. |
| Larsson & Hodgson 2023, USA. | American Indian children enrolled in Head Start program (n=475) in Northern Cheyene Head Start community, USA. | To determine the effectiveness of an interprofessional health team in improving access to oral health care among American Indian children enrolled in Head Start. Intervention study utilising nursing students, nurse practitioners, dental hygienists, and tribal leaders. | 313 cleanings, 442 fluoride varnish applications, 801 sealant placements or repairs, and 99 applications of silver diamine fluoride for 475 preschoolers were provided. There were 161 children referred for treatment of whom 123 completed treatments (76.4%; 59 boys). | No mention of cost of program but states funded through a combination of federal, state, and private grants. | Introduction of teledentistry component decreased time to care for children. | Delivery in community can reduce time to treatment from referral. | Service was designed to address the needs identified by the Tribal communities. Highlighted that interprofessional teams with members operating to their full scope of practice can provide missing pieces to the puzzle of access to dental care in rural areas. | Not evident. | Provision of letter to parents and oral health supplies for home and supervised twice-daily classroom brushing. |
| Larson et al., 2019, USA. | Patients accessing the Northern Dental Access Centre in rural Minnesota (20,367 clients) from 2009-2016. | To evaluate the Northern Dental Access Program. Evaluation design | Between 2009 and 2016, 20367 unique clients were seen in the clinic. The staff performed more than 307000 prevention and screening procedures, more than 55000 fillings and restorations, and 20000 oral surgery/endodontic procedures. Overall, 32% of patients (n = 6626) completed their treatment plans. | As a safety-net provider, 98% of patients are enrolled in Minnesota’s Medicaid programs, and procedures are limited to the dental benefit set available to children and adults, which are outlined in state statute | Service is free to Medicaid clients | Bivariate comparisons suggested that those who were provided transportation assistance (5% of all patients) were more likely to complete their treatment than the overall patient population. who had at least 1 clinical visit. Distance travelled appears to be a factor in treatment completion. | Mentioned cultural competence of those working there. | Authors outlined the importance of ensuring that the environment did not perpetuate stereotypes and confirm the distaste clients have for being there. They outlined that they purposefully created an environment filled with people who demonstrated the organization’s core values. | Referral for wrap around services but no specific mention of information provision to ensure patients were aware of service. Documents importance of consultation with a patient focus group to provide “reality therapy’ so that to ensure service meets patients needs. |
| Lee, et al., 2019, Taiwan. | 347 Aboriginal people with betel quid chewing or cigarette smoking experience. The participants were randomly assigned to intervention (IG; n = 171) and control groups (CG; n = 176). Remote aboriginal communities in Taitung in the east of Taiwan and Pingtung in the south of Taiwan. | To evaluate a health belief model (HBM) intervention using lay community health advisors (LHAs) for oral cancer screening (OCS) and mouth self-examination (MSE) in remote aboriginal communities. Randomised controlled trial | The IG participants were 2.04 times more likely to conduct a monthly MSE than those in the CG (95% confidence interval: 1.31–3.17) and showed significantly higher self-efficacy levels toward OSC and MSE (β = 0.53 and 0.44, effect size = 0.33 and 0.25, respectively) and a lower barrier level for OSC (β = 1.81, effect size = 0.24). | There was no mention of costs or if the LHAs were paid to carry out the trial but authors state that LHAs can help local health departments to save on staffing costs. | As LHAs are local people there are available within community. | Cultural and cost issues can be alleviated by using LHAs. | Outlined that each aboriginal community has a unique cultural background and that cultural differences and insufficient resources are major barriers to cancer screening. Using LHAs can assist with this. | The use of LHAs increases likelihood of performance of MSE. | LHAs in CG received just leaflets and the IG received face to face training and leaflets. |
| Lee, et al., 2017. South Korea | 42 rural Korean residents older than 40 years participating in an oral health care program. | To analyse the effects of an oral health care program administered at rural public health subcentres on oral hygiene status and bleeding on probing (BoP) scores among Korean rural residents older than 40 years. Intervention study. | The BoP score decreased by 22.87 in the intervention group and 0.27 in the control group between baseline and the eighth week (p<0.001). Multiple regression analysis showed that the change in BoP score (∆BoP score) increased significantly with an increase in the reduction of the plaque (PHP) index (∆PHP index) (t=–2.174, p<0.05) and increased significantly more in the intervention group than in the control group (t=2.143, p<0.05). | Highlights that rural residents often have lower SES. education, or incentive to engage in preventative oral hygiene practices. | Outlines that reduced availability of dental education and services in rural regions further limits access to quality and effective oral health care. | Prevention of periodontal disease can be achieved by having health professionals working at community health centres. | Not evident | Not evident. | Minimal detail of participants listening to a verbal description of the project. |
| Mangoyana et al., 2023, AUS. | 38 participants. Intervention is a partnership between a university dental school and an Aboriginal and Torres Strait Islander Community Controlled Health Service (ATSICCHS) in 2 North Queensland rural towns. | To explore the benefits of a partnership between a university dental school and a Community Controlled Health Service, specifically in relation to improving the oral health of an underserved rural Indigenous community. Descriptive qualitative study. | The Indigenous community representatives expressed positive benefit in both their general and oral health awareness, in improved access to dental care provided in their own safe space, while they were pleased to assist with students' learning. They viewed the partnership as mutually beneficial. Suggestions for enhancement of the oral health service were also offered. | Service provides free dental care to all patients no matter their financial status or identity. | Reported that improved communication and decreased wait times however the community felt communication with them about the clinic and its operation could be improved and expressed that there were still some barriers to accessing the clinic timely due to long wait times. | The model improved accessibility with transport services for patients from neighbouring townships implemented to facilitate access to services including the student clinic. | Engaging the Indigenous community as partners empowers and enables self- led design and delivery of services that improve equitable access to culturally safe oral health care as determined by Indigenous people themselves | Model was set up to provide culturally competent care. This successful model can also apply across different geographical areas because findings suggest that underlying experiences of Indigenous communities such as cultural safety and barriers to access transcend geographical regions | Community members engaged in all aspects of the research in a culturally safe way but no direct mention of information provision. |
| March, et al., 2023, AUS. | 9228 services over 3 years. Student led dental clinic. Two Indigenous communities in Southwest Queensland. | To determine whether oral health services provision by a dental student clinical outplacement embedded within a Community Controlled Health Service positively impacted a rural Indigenous community and to explore the nature of these benefits.  Retrospective analysis of student led dental clinic patient data from 2017,2018,2019. | The student-led clinic services mix shifted over time from mainly acute care for toothache towards prevention of disease and tooth restoration, indicating an improvement in patient oral health and correspondingly reduced system costs. Imputed value of 2017–2019 student-led clinic services provision totalled almost AUD$1 million. Government public clinic waiting list times decreased after full establishment of the student-led clinic, indicating decreased pressure on the public system. | Free cost to community but economic value of the university clinic service activity resides principally in easing pressure on an overburdened public oral health sector by freeing up appointment times for other eligible clients. Assigning a monetary value to services provided by the student led clinic was notional as all treatment provided was free of charge and generated no revenue. | Available to all within the community (health care card or not). | The student outplacement clinic drove an improvement in access to and timelier delivery of care for the Indigenous community. | Services delivered with cultural sensitivity, which has spill-over benefits for their work in boosting community oral health awareness. | Not evident. | Integrated oral health clinic delivered in culturally sensitive way. No specific mention of information provision. |
| Masoe, et al., 2014, AUS. | Adolescents (12 to 18 years of age), New South Wales, Australia accessing the NSW Public Oral Health Service. | To record the proportion and type of preventive care and clinical treatment activities provided by Therapists to adolescents accessing the NSW Public Oral Health Service.  Retrospective data registry study. | Therapists responsible for 79.7% of preventative care and 83% of restorative treatment offered to adolescents accessing Public Health Oral Services over one year period. Preventative care provided by therapists for adolescents varied across Local Health Districts ranging from 32% to 55.8% of their clinical activity. | Adolescents in New South Wales are eligible for free oral care until their 18th birthday | In 6 rural LHDs Therapists provided lower levels of dietary advice to adolescents compared to Metropolitan areas. | Rural and remote Local Health Districts (LHDs) undertook less preventative care (45.2%) compared to metropolitan counter parts (51.6%). Overall, rural areas provided lower levels of dietary advice (below 10%) compared to metropolitan areas. | Rural and remote Local Health Districts (LHDs) undertook less preventative care (45.2%) compared to metropolitan counter parts (51.6%). Overall, rural areas provided lower levels of dietary advice (below 10%) compared to metropolitan areas. | Not evident. | Refers to advocating for more oral health promotion resources but limited details. |
| Mathu-Muju et al., 2016, Canada. | Children’s Oral Health Initiative (COHI) that targets preschool children; 5–7-year-olds; pregnant women; and parents/caregivers in First Nation and Inuit (FN/I) communities in Canada. | To increase access to oral health services provided to First Nations and Inuit (FN/I) children living on federal reserves in remote communities. Long-term outcome to decrease levels of dental disease in FN/I communities. No clear method described. | Approx 50% of FN/I children participated in COHI program across Canada - see Table 1 for regional breakdown. 222 COHI aides recruited by 2013. High participation rates in the program within COHI communities, demonstrating COHI is a viable preventive oral health care delivery model. | N/A - Federal government support to set up program. Not applicable to individual client pricing. | Sustainable model supported by community engagement and commitment to the model- social capital model to sustain and implement. | COHI has been shown to be a delivery model that has improved access to preventative oral health. | Program supports FN/I autonomy in managing health services to meet the needs of the communities. | Not evident | The development, training and utilization of a community health worker to promote and support oral health activities in the community is a significant innovation of the COHI initiative |
| Mathu‐Muju, et al., 2018, Canada. | Sample size: n = 25 (F/NI) from the Children’s Oral Health Initiative (COHI) program in Manitoba, Canada. | To investigate effect of the availability of local community health workers (COHI aides) on access to COHI programme preventative dental services to children (in remote Canadian indigenous communities). 25 communities continuously enrolled into COHI program over 7-year period. Longitudinal evaluation. | Children in communities with uninterrupted service tend to have higher rates of enrolment and service delivery. Community health workers were beneficial in promoting program enrolment as well as facilitating and augmenting the delivery of preventative dental services. over 7-year period there was a cumulative total of 21,174 enrolments in the program. | N/A - Federal government support to set up program. Not applicable to individual client pricing. | COHI's success in building community capacity with local people helps support the advance of Indigenous communities towards autonomy in health governance. | Community oral health workers were beneficial in promoting programme enrolment and augmenting delivery of preventative dental services. | Study finding demonstrate the effectiveness of Indigenous community health workers in encouraging enrolment in an oral health prevention programme, which is a critical first step in sustaining the programme over time. | Not evident | Not evident |
| Maurizio, et al., 2003, USA. | Public aid recipients undergoing 702 examinations in the Heartland Dental Clinic in Southern Illinois University, Carbondale (SIUC) dental hygiene clinic facility, Jackson County, Illinois, USA. | To provide comprehensive oral healthcare to underserved populations (inc. rural) through a Public Aid Clinic in an educational dental hygiene facility.  No clear research method. | The developed Heartland Dental Clinic model is a cost-effective method for increasing oral health access to underserved populations whilst benefiting dental students in practical education and skills development. | The program uses existing educational dental facilities to address issues of affordability and access to dental care - providing a public aid clinic to underserved, underprivileged populations. Adults are ineligible for preventative care under Illinois Medicaid rules but can receive inexpensive preventative care through the dental hygiene clinic in a fee-for-service arrangement. | Provides a service that would not be available if model had not been instituted. Resource constraints for the program have been identified, as well as transportation issues. Lack of transportation is a major access-to-care issue for low-income people, particularly in rural areas. Access is interrupted during some university breaks. | Model provides increased access for SIUC area where local needs assessment in 1999 indicated 42% of low-income residents received no oral health care, yet 83% indicated they would use an oral health clinic if available. 702 examinations provided to public aid recipients over a 2-year period. | Allowing dental hygienists to take equipment to various facilities and perform preventative procedures would enhance access to care in rural communities. | Not evident | Not evident |
| Mays & Maguire, 2018, USA. | 43,128 dental patients (adults and children). 418 student providers. to 2 extramural clinics and the mobile dental clinics and public health clinics in Minnesota State from May 2013 to April 2016 at 11 clinical sites. | To develop a 3-year profile of patients seen and care provided by students at extramural clinics in one U.S dental school. Retrospective data analysis of dental school database (over 3 academic years) of community-based dental education (CBDE) clinic program. | Three student cohorts participated in rotations. Over the 3-year period 43,128 patients were treated by 418 student providers. Approx 25% of all encounters were with pediatric patients. Students completed 5,908 child prophylaxis, 5,386 topical fluoride varnish, and 7,7678 sealant procedures on pediatric patients. 7% of total patients treated has special health care needs. | Clinics were provided to underserved populations receiving Medicaid benefits. A total of $16,614,680.36 worth of care was provided during the three years in the state of Minnesota Report suggested that future studies should document the impact of providing care at extramural sites and defining financially sustainable models. | The provision of care by students as part of the program with the clinical rotations primarily established to address the Commission on Dental Access Accreditation (CODA) predoctoral standards but also serves a secondary benefit of providing safety net care for an underserved population. | The students in the program are helping to provide access to oral health services in underserved areas. | 47% of patients cared for by students as part of the program were from underrepresented groups. | The program positively improves student's experiences and attitudes about providing care for populations that are underserved and receive Medicaid benefits, and how student apprehension about providing care at extramural sites can be managed. | Not evident |
| Meihubers, 2013, AUS. | Children 0-5, young mothers/carers, school-aged children and people with chronic disease participating in the 'See my Smile' program in Aboriginal Bila Muuji communities in New South Wales (NSW), Australia | Collaborative approaches to community-based oral health programs. Aim to identify and implement oral health promotion priorities and activities across the Bila Muuji region.  Evaluation design. | Profile of oral health raised and coordination with other health and community program areas has increased. | Not evident | Acceptance of the program and growth in communities has been driven by the inclusion of local staff from the beginning, and the active participation of partners such as the Local Health District. | Acceptance of the program and growth in communities has been driven by the inclusion of local staff from the beginning, and the active participation of partners such as the Local Health District. | The regional oral health promotion program formed an oral health partnership with Bila Muuji representatives and was co-designed and co-led to meet their needs. | Report stresses the importance of working with local staff meaningfully. Inclusive design and involvement of local people in program from the start with workshop participants given opportunities to share information on oral health and related issues in their communities. | Working with ACCHO staff in each location, the regional coordinator provided oral health information and resources relevant to these groups on a regular basis and also supported the ACCHO staff to take the lead in future programs such as school-based toothbrushing programs. |
| Milgrom et al., 2013, USA. | Pregnant women aged 15 or over. English speaking and eligible for Medicaid (Oregan Health Plan Plus) in four rural communities in Oregon State, USA. Sample size: 400 women with 80 to 120 women enrolled within any one county. - session 9 months postpartum with the focus on the child. | To explore dental utilisation during pregnancy or up to two months postpartum for mother, and preventative dental utilisation by 18 months of age for the child. Five-year, four-site randomized intervention trial with a 2 × 2 factorial design. 4 treatment arms. Medicaid data used to assess primary outcomes. Questionnaires administered to participants at enrolment, and 3, 9 and 18 months postpartum. Trial design aimed to increase low-income pregnant women's utilisation of dental care, increase young children's dental care utilisation, and improve home oral healthcare practices. | Paper is a protocol for the design of the intervention | Trial designed to increase low-income pregnant women's utilisation of dental care. | Not evident. | Choice of setting allows Baby Smiles trial researchers to reach low-income women who were unlikely to have a usual source of dental care. | Not evident. | Not evident. | Included a health education intervention including video and pamphlet material. |
| Ng, et al., 2024, New Zealand. | 100 residents and 77 care staff from 14 aged residential care facilities in the Otago region of New Zealand | To estimate the level of acceptability (perceived benefits and comfort) of using teledentistry among aged residential care (ARC) residents and staff in the Otago region of New Zealand.  Cross sectional survey. | Three- quarters of resident participants thought that teledentistry was beneficial. Staff participants were receptive to teledentistry use for residents and were comfortable facilitating remote dental consultations and care through teledentistry. No staff participants disagreed with the potential benefits of teledentistry for ARC residents. | Free text comments from participants cited that teledentistry would reduce cost and travel expenses. | Free text comments from participants outlined time and energy savings of teledentistry | Participants thought it would be more convenient | Less pressure on ARC staff and family members | Measured by perceived benefits and comfort, was lower among older participants | Outline of importance of information provision as part of teledentistry but no detail of what was provided outside of training for intra oral cameras |
| Nycz, et al., 2020, USA. | 171,293 dental patients’ resident in rural Wisconsin (north, central and western areas), USA. | Increasing dental care access and reducing access disparity of oral health services in rural Wisconsin. Programs strategic aims: (a) build multi-practice dental centres responsive to access need; (b) achieve medical-dental integration, and (c) accommodate special needs patients. Community Action Report/Blueprint of a community health center (CHC)-based program targeting reduction in access disparity. | Program's investment greatly expanded dental care access for disparity populations. Program has improved staff recruitment and retention, supported mentoring for less experienced staff, provided training opportunities for fourth year dental students, helped maintain patient service levels during periods with staff shortages. Increased dental access led to reduced use of emergency department (ED) hospital services to treat non-traumatic oral health problems | A key dimension described in the blueprint identified covering patients with specific needs including publicly insured and poor/near poor uninsured. 85 % of patients are Medicaid and uninsured (dental) low-income individuals. | Program used patient origin maps using patient data to determine care-seeking patterns to identify communities with highest need. These initiatives have substantially contributed to regional access disparity reduction. | A multi-site initiative to be improve access to dental services. The dental initiatives have substantially contributed to regional access disparity reduction. | The program was designed to accommodate special needs patients. | Feedback from urgent care and emergency room physicians linked the dental centre establishment with reduction in non-traumatic dental emergency visits and opioid prescribing. | Not evident |
| Pacza et al., 2001, AUS. | 27 x Aboriginal Health Workers (AHWs) serving total populations of 16,100. Kununurra (4,800) and Broome (11,300) Western Australia (WA). | To institute a culturally appropriate basic preventative oral health delivery program at a community level and improve community oral health through a dental training program for Aboriginal Health Workers (AHW)s.  Evaluation design. | 27 AHWs completed 2 modules of a pilot training program and a general questionnaire. Smaller random sample of students from Broome completed a detailed questionnaire (evaluation of the training program). Program provided AHW participants with a moderate knowledge of conservative and preventative dental treatment modalities, relevant to rural and remote client groups. | Not evident. | Not evident. | Training program tailored to the needs of rural and remote AHWs to assist in implementing long-term preventative measures at a local level to improve community dental health. | Training program which is culturally adapted to meet client needs. | 96% of students considered training material relevant to their needs. | Detail of information provided in the training modules. |
| Patel, et al., 2023, AUS | Aboriginal and Torres Strait Islander communities in remote Kimberley region of Western Australia (WA). | To describe a non-government, volunteer organisation designed to fill service gaps and extend dental care to areas of unmet need.  Evaluation design. | Ten years of service model. Now well established including mobile dental units. 43 schools in toothbrushing program since 2014. Have seen a 3% linear reduction in emergency dental extractions since 2013. Now primarily preventive and restorative treatment rather than emergency surgical care. | Daily school brushing program designed to offset the high cost of fluoridated toothpaste. 20,000 oral hygiene packs distributed to schools. Services are provided pro bono from volunteers. | Annual dental check-ups by the detail team. Volunteers only need to commit to 1 week. Transport and accommodation costs are largely supported by the program. Hub and spoke model. | Now includes mobile vans to increase access. | A teaching guide and culturally appropriate educational material was developed. Recyclable canvas bags hold children’s oral health supplies enabling them to personalise them. | Craig the crocodile is a mascot that encourages kids participation. | Outlines strong links to community but not specific about information provision. |
| Patel et al., 2021, AUS. | Aboriginal adults, 23 interviews and 17 yarning groups (N=80). Remote dental service. East Kimberley region, Western Australia. | To investigate the perceptions and attitudes among aboriginal Australian families living in Kimberley toward dental services. Qualitative study. Semi structured interviews & yarning circles. | Enablers to care included: provision of culturally appropriate care the use of mobile dental services, heightened awareness of existing services and supplemental services provided by visiting volunteer teams. Barriers included limited transport, affordability of services, the complexity of appointment systems, dental anxiety, practitioner experience, hospital-based treatment, shame associated with oral health and health seeking behaviours, and the impact of social determinants on oral health (e.g. housing, poverty and transgenerational trauma). | Not evident | Operated by dental health service currently with clinics in larger towns. Outreach services to remote communities but frequency variable. Volunteer services 'fill the gap'. Kimberley dental team established in 2010 - voluntary treatment a no cost. | Lack of transport to nearest dental clinic biggest barrier to care remoted by all Ps. | Volunteers seen as a stop-gap measure. Long term solution needed. Lack of publicly funded dental care at odds with public medical care. Need to schedule appointments and complete pre-appointment paperwork a barrier. Need a walk in service. | Concept of shame and being judged. Central to willingness to seek care. | Aboriginal liaison officers supported the recruitment and management of the study. Information provided in culturally appropriate ways. |
| Patel, et al., 2015, AUS. | Volunteers with the Kimberley Dental Team (KDT). N=42 Volunteer program. Kimberley region, Australia. | To describe the demographic profile of volunteers, identify their attitudes and values, and explore their perceptions regarding barriers and enablers for good oral health for Aboriginal Australians. Structured online survey | Mean time volunteering was 2.4 years. Many had worked oversees and in rural/remote regions. Background in clinical dental practice. Give back to the community and to help disadvantaged. Biggest barrier to volunteering were time constraints. Oral health was perceived as poor. | Not evident | Not evident. | Not evident | KDT had raised awareness and improvement seen in areas visited by KDT. | Not evident | Volunteers outlined the importance of culturally appropriate services and need for better services but little detail of information provision. |
| Pawloski, et al., 2022, USA. | Medical and dental providers and clinical administrators in the medical-dental integration program (N=12). In primary health care settings in Eastern Washington State, USA | To elicit perspectives and experience of providers and administrators involved in the MDI program to assess the acceptability, feasibility and success of an MDI integration strategy.  A qualitative program evaluation. Semi structured interviews | Three key themes: the MDI program is feasible and acceptable, implementation faced systemic and behavioural barriers, and the program is perceived as beneficial to parents and successful for the CHC. | The program was in the context of a well-child visit at 2 paediatric medical clinics. Dental services were not billed out separately. Medicaid would not reimburse separately. | Volunteers thought lack of access to care was greatest barrier. | Integrated with medical clinic so presumably easier but the study was not about patients’ perspectives (but providers). believed it would catch patients falling through the cracks. | There are advantages of the dental hygienist as the integrated care provider embedded in medical clinics. Hygienists seen as ethnically congruent with patients and fluent Spanish speakers. | Not evident | Consistent, adequate communication with leadership was key to reduce perceived pressures of workflow changes |
| Piggott, et al., 2021, AUS. | Parents and cares of Aboriginal children in RCT of ART and Hall Technique (N=29, 10 from intervention communities and 19 from controls) in an Australian community. | To elicit the experiences and perceptions of parents and carers who participated in a project that tested the minimally invasive atraumatic restorative treatment (ART) and the hall technique (HT) approaches to manage early childhood caries among aboriginal preschool children. Qualitative evaluation of a cluster community RCT of ART-HT approach with 12-month follow-up | Three main themes; access to care (barriers, service availability, impact on family due to lack of access); experience of care (cultural safety, child-centred care, comprehensiveness of care); community engagement (service information, engagement, oral health education). | Not evident | Availability of services is limited. Infrequent visits and limited time by providers in the community. | Limited access to vehicle, financial demands and unfamiliarity of larger towns, time away from school and work paced stress and burden to attend. Cultural safety when navigating unfamiliar environments and processes. | Unclear and confusing bureaucratic processes of eligibility criteria, forms, waitlist appointments and fees were barriers to care. Parents confused about age at which children were eligible. Minimally invasive approach well received. | Information about treatment needs and regimes when given in lay terms reinforced the feeling of trust and collaboration with parent/carer. Other treatment providers had resulted in shame/guilt. Non-judgemental information important. | Parents and carers spoke of the value of strong engagement with the community and of receiving clear and timely notification of service provider visits by the project team. This included flyers, signs, door knocking. This developed trust in service. |
| Ragade et al., 2023, Australia. | Pregnant women, 18 years of age or older, eligible for public dental care in a rural health service in the Loddon Mallee region, Victoria, Australia, 62 women were referred into the service and 44 received dental care. | To analyse the effectiveness of targeted stakeholder engagement strategies and the impact they have on antenatal referrals and oral health admission of pregnant women to a rural publicly funded dental clinic. A quasi-experimental quantitative design was used. Participants were grouped as either the pre-intervention or post-intervention group, depending on when they interacted with the key stakeholders.  Pre/post design. | Active engagement with key stakeholders significantly increased the oral health referral, admission and attendance of eligible antenatal women. Prior to the intervention only 15.04% of eligible antenatal women sought oral health treatment compared with 40.37% post intervention. | Antenatal women are in possession of a concession card for priority and immediate public dental services. However, about two thirds did not have a concession card. Intervention was delivered in private clinic too. | The number of preventative treatments increased from 20 to 96. Number of restorations completed increased from 20 to 79 and number of extractions increased from 15 to 22. No suggestion of unmet needs. | Increased accessibility because stakeholders were from a range of services in the local catchment area. | The results from the study indicate that raising awareness among healthcare workers is an effective means of increasing oral healthcare engagement in a rural setting, but there is no feedback from the clients on their individual needs directly. | Not evident | Midwives provided with an education session and mothers were provided with a range of oral health information. |
| Rajabiun et al., 2011, USA. | Dental and program directors of dental services for people living with HIV/AIDS (PLWHA). 15 programs and 45 staff from across US including AIDS service organisations, community health centres, university-affiliated medical centres or hospitals. 5 (33%) were rural, 6 in major cities, and 4 served both urban and rural. | To identify the key elements that may be used by organisations for designing programs to reduce barriers and expand dental services for PLWHA and other underserved or vulnerable populations. 3 sources of data: 1) site visits to 15 programs including semi-structured interviews with 45 staff. Self-reported data on dental services lists. Follow-up phone interviews with staff. Focus group exercise at a semi-annual meeting with program and dental staff. | 6 main categories emerged as key elements to deliver dental services: the type of dental services provided, dental care coordination for patients, professional training for future dental providers, transportation assistance and other ancillary services, focussed patient education, and connections to medical care. | Not paid for by client. | LWHA oral health care is most reported unmet need. No evaluation of whether the programs did this or not. Rather just a reporting of a typology of programs. | In general, mobile dental services provided lower-level services across all sites, and higher-level services were provided through collaborative relationships with other providers in the community. Some offered transportation services to patients - key element for engaging and retaining patients. | Dental care coordination in several programs to support the patient access, attend and follow-up dental services. Doesn't say if this is successful from patient perspective. | Patient education at chairside or before appointment or in waiting room. Again, no information on whether effective. | Programs used individual and group sessions to educate patients about manifestations of HIV in the oral cavity, importance of dental care and oral hygiene practices. |
| Ramos-Gomez et al., 2024, USA. | Workshops were conducted with a total of 451 residents  (276 teachers/staff and 175 parents) by UCLA paediatric dental residents in California USA | To assess whether staff,  teachers and families from rural/migrant Head Start/Early Head Start (HS/EHS) programs in  California was receptive to oral health online education workshops conducted by paediatric dental  residents who were assisted by bilingual (English and Spanish) community oral health workers (COHWs). Formative research design with process evaluation. | Partnering paediatric dental residents with bilingual COHWs to  educate HS/EHS teachers, staff and parents on oral health care in rural/migrant areas could result in  a rewarding experience for paediatric dentists that might lead them to practice in these communities  upon graduation from their residency program. | Costs for early education and childcare for low-income families. However, it remains unclear if this covered the cost of the workshops. | Development of health-related topics were of more interest to organisations based on the needs of the total population. For some residents not all content was delivered in the workshops. | Workshops delivered on Zoom. Access enhanced by greater awareness of rural/migrant areas during dental residency training and use of bilingual COHWs. | Many of the participants joined the oral health workshops from home or at a HS/EHS facility. | No reported demographic data (such as age, gender, ethnicity). However, process evaluation contains information from educators such as Spanish delivery as being more meaningful to communities. | Not evident |
| Roberts, et al., 2016, USA. | RIDE program. Students spend about 40% of their time in communities away from dental school. Exposes students to a public health model of care.  Eastern Washington State, USA. | To deliver intensive community-based education that prepares dentists to meet the needs of rural and underserved populations including rural elders. Evaluation description. | 70% RIDE graduates have settled in rural or underserved communities in the region. | No detail but RIDE is seen as a cost-effective model for delivering graduates to region which have a need for dentists | Not evident | Not evident | Not evident | Not evident | Not evident |
| Roberts-Thomson, et al., 2010, AUS. | Indigenous preschool children (N=30 communities; 666 eligible children examined at baseline. Communities in Australia's Northern Territory in PHC settings. | To evaluate the effect of a community orientated primary health care intervention on the oral health of indigenous preschool children living in remote communities in Australia's Northern Territory. Community clustered randomised controlled trial. Communities were randomly assigned to intervention and control groups and all children from a community were in the same group. | Almost all children (89%) developed new caries over the 2 years. A similar proportion of children in the intervention reported having a sugary drink in a similar ratio to baseline. The % reported tooth cleaning increased to 40% in each group. No difference in clinical measures between I and C groups. | Not evident. | Not evident. | Not evident. | Got a good uptake for the intervention. | Found in difficult to engage primary health care staff to be involved in delivering the intervention - too difficult to accommodate with heavy workload. Need a dedicated staff member for oral health. Need to integrate within broader primary are activities. | A number of activities to raise awareness including face painting for children, resources for parents and training for other health workers to raise awareness of oral health. |
| Rowland, et al., 2016, USA. | Rural Maryland population claims data (N=1600 unique clients/individuals across 2700 emergency department (ED) visits. | To assess the impact of the community dental access program (CDP) providing urgent dental care to low-income individuals on adult dental visits to a regional ED. Analysis of existing data from 2 sources: 1) CDP claims database and claims data for dental-related ED visits. For 2011-2015. | Almost exclusively seen for urgent care - extractions (32% of procedures) and restorations (33%). Over 2011-2015 over $1.7 million in dental services were provided. Providers donated 69% ($1.17) of total costs and CDP reimbursed the rest. Visits to ED decreased from 2011 to 2015 from 1500 visits to 1100. ICD-9 diagnoses decreased from 1800 to 1500. A marked decrease in dental-related regional visit to ED in 2015 compared to 2011. | Clients didn't pay. CDP paid alongside providers. | Unclear although did lead to substantial reduction in use of ED. | Not evident | Unclear although led to less ED visits, and more emergency treatment. Delivery of care relies on grant funding and on willingness of dental professionals to discount typical rates and see patients who cannot afford dental care. | CDP subsidized by grant funding and willingness of dental providers. Sustainability an issue. | Not evident |
| Schroeder, et al., 2021, USA. | Adults at a rural farming exposition in Wisconsin USA (N=236). N=193 participated in oral cancer screening. | To collect information related to oral cancer awareness, offer education and provide oral cancer screenings at an educational farming event in the state of Wisconsin. 12-item survey and a visual/tactile head and neck examination/oral cancer screening. | 65% correctly identified two or more signs of oral cancer, with 19% identifying one. Oral lesions were found in 17% (N=33) with 52% male. 70% (N=23) with visible lesions reported having seen a dentist in the past 6 months and 36% had dental insurance. | Patients sought routine preventative oral health care regardless of insurance status. However, they lacked knowledge of oral cancer risk factors, signs, symptoms. Some who had seen a dentist regularly had lesions. | Suggests that interventions beginning in secondary and high school and continuing into other educational levels might be effective preventative care strategies. Also need preventative head and neck exams in routine oral health are visits. | Preventative check-ups might not be happening in routine dental visits. | Head and neck examinations in community event settings may reach some in rural farming communities but not all. | Not evident | An informational poster board displayed at the screening both and other printed materials for participants (eg quit smoking, signs and symptoms of oral cancers). |
| Senturia, et al., 2018, USA. | Interviews with community members (n = 19; healthcare workers (n = 19); FGs: patients (n = 31 adolescents and 16 caregivers of children under 12 years) living in or providing health care to 3 remote villages in Alaska. | To describe paediatric dental care for children living in remote villages in Alaska from the perspectives of the community and the healthcare providers. Qualitative study: semi-structured key informant interviews (purposive sample of community and healthcare organisation stakeholders) and Focus group discussions (convenience sample of patient stakeholders). | 19 community leaders and 19 staff, some were also community residents, participated in the key informant interviews; and a combined 47 adolescent patients and caregivers of paediatric patients participated in the focus groups. Two major themes emerged: PDHAs and DHATs are in a strong position to meet the unique needs of remote Alaskan villages, and they are sustainable. Second, PDHAs and DHATs face barriers that limit their effectiveness. They are not always well utilized and require better management and role clarification. | Unclear but did discuss staff efficiency and productivity. | Staff training has been provided - but staff underutilised and roles ill-defined. | Examined the potential for better integrating specific health care roles - in rural villages with unmet dental health care for children. | Identified barriers and gave suggestions for better delivery of care to meet need. | Focused on the local context and the healthcare system needs and capacity to reduce oral health disparities for Alaska Native children living in remote villages. Data confirmed the acceptance of both the primary dental health aide (PDHA) and the dental health aide therapist (DHAT) by the care system and the rural communities and identified key barriers for future quality improvement efforts to focus upon. | Outlines importance of one-on-one sessions and culturally appropriate information. |
| Shah et al., 2024, USA. | Dental records of 1168 children were reviewed. 50.3% were girls and 49.7% were boys from a rural paediatric population in the Western region of New York, USA. | To evaluate the synchronous teledentistry program that  was established in 2010 regarding adherence to dental treatment recommendations of more than  1,000 paediatric patients from rural areas of Western New York.  They hypothesised that the synchronous teledentristy program considerably improved treatment completion among underserved rural children compared with treatment completion rates before initiation of the program. A retrospective review of dental records. | Nine hundred fifty-four  children (81.6%) completed the recommended treatment within 6 months of synchronous teledentistry  consultation. Treatment completion rates varied significantly according to treatment  modality. In-office consultation had the highest rate (96.8%), followed by oral sedation (89.7%)  and operating room treatment (89.5%). Nitrous oxide (66.7%) had the lowest rate (P < .0001). | Children involved in the study had New York issued dental insurance including Medicaid of Child Health Plus. | Synchronous teledentistry consultations with rural paediatrics patients effectively facilitated treatment completion in underserved areas. | Increased access reported. Teledentistry improved access to speciality oral health care, reduced treatment time and costs and enhanced oral health outcomes for underserved paediatric dental patients. | Study reports on complex needs in paediatric populations and appears to address these needs. | No race or ethnicity data were available in the reviewed records. Sex was reported. | Not evident |
| Shimpi et al., 2023, USA. | Patients who sought medical and dental care in rural Wisconsin, USA. During the 30-year study 21,957 patients made 34,892 documented visits to medical centres. | To examine the impact of expanded dental access on reducing patient attendance to medical centres for preventable infectious dental diseases (PIDD), pre and post implementation of regional dental centres.  A retrospective observational study. | In this study, greater access to dental care appeared to reduce PIDD visits to medical settings.  PIDD visits in primary care settings was 0.87 times as likely as PIDD visits at ED/UCs after dental centres opened. | Lower fees reported for low-income and underserved individuals. The dental centres have sliding fee scales that permit treatment at partial or no cost, based on federally defined poverty status for those 100% to 200% of the Federal Poverty Line. | Greater availability reported, in part due to integrated care delivery system and lower fees | Medical centres were established to reduce dental access disparity with the intention of providing affordable and accessible dental healthcare for low in-come and underserved individuals. | The study reports on dental disorders, dental caries, gingival and periodontal disease, periapical abscess/pulpitis and loss of teeth (other than from trauma). Addresses a range of dental needs. | Age, race, gender, ethnicity and funding source reported. | Not evident |
| Siegal, & Detty, 2010, USA. | 11,118 records included in the analysis. Total initial sample = 14,025, representing a population of 127,194. Third grade school students in Ohio, USA. State-wide survey captured schools with and without the intervention. A total of 374 schools were selected from among 1,960 schools. | Aim was to evaluate the intervention. A stratified, clustered random sample was drawn from a listing of schools with third grades in Ohio. | Results: At schools with no S-BSPs, higher risk children were less likely to have dental sealants than lower risk children (28.7 percent versus 42.7 percent, P < 0.001). At schools with S-BSPs, sealant prevalence for both risk categories was equivalent for higher and lower risk children (59.4 percent, 63.4 percent, P = 0.428). Higher risk children at schools with S-BSPs were more than twice as likely to have a sealant as higher risk children at non–S-BSP schools (59.4 percent versus 28.7 percent, P < 0.001). | S-BSPs are most often funded with public dollars and operated by public agencies or private nonprofit organizations, and sometimes by educational institutions. Central to the purpose of S-BSPs is serving higher risk children, including those less likely to receive private dental care. | Intervention schools: The Ohio targeting standard for state-funded S-BSPs in 2004-2005 was 50 percent FRPMP enrolment for urban schools and a median income of less than or equal to 150 percent of the Federal Poverty Level (FPL) primarily for rural school districts. The effect of these criteria was a statewide program that, in the 2004-2005 school year, served 464 schools of which 84 percent had 50 percent and 93 percent. had 40 percent of second grade students enrolled in the FRPMP. | Findings demonstrate that these programs are effective in reaching their target population of higher risk children. | Not evident | Not evident | Not evident |
| Skelton et al., 2009, USA. | Pregnant women accessing pre-natal care. During 2006 and 2007 447 women were enrolled in the programme. A rural health care clinical in Western Kentucky - oral health integrated into group pre-natal care package. | To report the evaluation of an intervention integrating oral health care and education into group pre-natal sessions.  Pre and post analysis of data collected at the individual level. | Of the 447 women enrolled, 379 had given birth by the time of writing of the article. Positive outcomes - i.e. mutual learning and respect between different health professionals reported. Over 4/5 on public assistance for medical care and had completed no more than high school. Initial exam (13-16 weeks gestation): oral health status ranged from extremely poor to very healthy. Nearly 70%had active caries over 50% had at least one periodontal pocket of 4mm or more and 1/5 had pain in one or more teeth. Over 17% had active pericoronitis infections and 16% periapical accesses. Over 60% had bleeding indicating gingivitis. | The degree of poverty among the mothers was very high (80% were on Medicaid). | Integrated into pre-natal care that mothers were attending. | Delivered at health centre in rural area. | Delivered at health centre in rural area. | Some issues with compliance and missed appointment mentioned. | Oral health information delivered in sessions |
| Skinner, et al., 2020. AUS. | Aboriginal children (5-12 years) in the primary school setting. Six ACCHSs from regional NSW, AUS | To investigate the feasibility of using Aboriginal dental assistants to provide regular fluoride varnish applications for Aboriginal children in the primary school setting.  Quality improvement study. | In total, 8 Aboriginal dental assistants were trained to apply fluoride varnish during the study, 6 in 2017 and 2 replacements in 2018. Results showed that Aboriginal dental assistants are able to safely and effectively apply fluoride varnish in a school setting with remote supervision. Lessons learnt: This program can be scaled at the state level in NSW, and this could provide the basis for a nationally consistent program. | Cost of the intervention came from the grant. | Delivered directly in schools. | Paper demonstrates an approval process in New South Wales for dental assistants to apply fluoride varnish. Dental assistants can safely apply fluoride varnish on a routine basis in primary schools in New South Wales. Concludes that scale-up of fluoride varnish programs for Aboriginal children in New South Wales is likely to significantly improve the oral health status of Aboriginal children. | Relatively low program enrolment percentages in some schools. Overall, the program enrolment percentage was 40%. Generally, schools that engaged an AEO in the consent process achieved a far higher consent response rate. | The consent rate varied from 30% to 90%. Five out of the 7 schools engaged an Aboriginal Education Officer (AEO) to liaise with families and consent children into the study. In the schools that engaged an AEO, the average consent response rate was 59%, compared to 42% in those schools that did not | Aboriginal liaison officer enhanced communication |
| Spetz, et al., 2019, USA. | Quantitative component: 84279 unique patients over 15-year period (2000-2015) Qualitative: 8 interviews completed with programme leadership, community partners and staff. A large mobile dental care programme in Minnesota. USA. | To evaluate a large mobile dental care program. Mixed methods Evaluation: thematic analysis of interview data collected during a 2-day site visit and multi-variate regression of electronic patient records (adults and children) that received care from 2000-2015 (84,279 unique patients). | Quantitative Results: Number and characteristics of patients served: CDS served 5558 unique patients in 2000 and 13,863 patients in 2015. Preventive services accounted for 45.7% of all procedures in 2000 but declined to 29.4% in 2015. Diagnostic services also declined as a share of all procedures, from 37.1% to 31.3%, while restorative services and adjunctive services increased. | Service specifically targeted to those who were un/underinsured. | Study identified shortage of providers - in general and dentists specifically as an important impediment to sustainability and growth pf programme. | Children’s Dental Services manages the distribution and collection of parental consent forms and determines the number of providers sent to a school based on the number of consents received. This results in varying penetration of the program within school sites. | This evaluation was limited to one state, and the following factors facilitated the success of the programme: expanded scope of practice of dental assistants, relatively high Medicaid reimbursement rates, and the licensure of DTs—that do not exist in many other states. These unique attributes limit the generalizability of the findings to other states, but also provide important guidance for other states and programmes seeking to expand oral health care access. | Engagement largely influenced by support of school staff (se accessibility). | Not evident |
| Stewart, et al., 2022, USA. | Children attending all elementary and middle schools in Bertie County. Public School district, USA. | Outlines of a community-academic partnership - formed to develop and implement a school-based oral health prevention programme. Used the PRECEDE-PROCEED model to develop, implement and evaluate a new intervention, developed collaboratively. Article is shaped round the 7 (P-P) phases of intervention development through to impact evaluation. | In the initial 4 months of program implementation, 138 elementary school children were examined. Approximately 82% of the children were enrolled in Medicaid, and 35% had active dental decay, with some having urgent needs such as pain or swelling. Findings suggest that parent/guardian perceptions often do not align with a child’s oral health status and that strategies are needed to improve parent/guardian awareness of their child’s oral health. | Cost of the intervention came from the grant. | Delivered directly in schools. | Intervention developed with a community-academic partnership - to make sure it was appropriately tailored. PRECEDE-PROCEED steps involved: | Community academic partnership | Data from parents/guardians and school staff were collected to assess their satisfaction with the oral health program. | Mentions low levels of health literacy and the need for information provision that is suitable. This included a ‘report card’ sent home to parents about their child’s oral health. |
| Surdu, & Langelier, 2020, USA. | 144 (74 girls and 70 boys) children experiencing serious dental decay - who experienced tele dentistry between 1 Jan 2015- 31 Aug 16 at one of several local dentistry clinics. Rural upstate New York. | To evaluate factors influencing utilisation of follow-up oral-health services  in general dentistry clinics among children after a teledentistry consultation and treatment with a paediatric  dental specialist. Appears to be an observational, cross-section study, but not explicitly listed so. | Most children completed a treatment plan (97.2%) at the specialty clinic and subsequently accessed follow-up oral health services at one of the local general dentistry clinics (77.1%) where teledentistry services were provided. Children's utilization of follow-up services in general dentistry clinics was associated with a shorter time to specialty treatment. | Not evident | Teledentistry provided within a specialist paediatric consultation. | Limited number of study participants diminished statistical power. But study suggests teledentistry consultations promoted access and utilisation of specialty oral health care. | Teledentistry provided within a specialist paediatric consultation. | Not evident | Not evident |
| Trudnak, et al., 2018, USA. | Underserved children and youth. 9 sites in rural USA. | To conduct and an independent evaluation investigating program efficacy, integration, and sustainability. Mixed methods evaluation of a school-based comprehensive oral health service. 3 domains measured: Efficacy; Integration and Sustainability. | A great need for comprehensive services for students demonstrated: on average, 45% had dental caries at enrolment. Enrolment increased from 5000 to more than 9700, and the percent receiving preventive services increased from 58 to 88%. Statistically significant increases in the proportion of enrolees who had their teeth cleaned in the past year and those receiving overall preventive services. Percentage of enrolled patients who completed a treatment plan within 1 year also increased from 37 to 63%. | Delivered in schools. | Only 9/12 sites included. | Rolled out across several USA sites. Delivered in schools. | Sustainability achieved - as still in operation 6 months after end of dedicated grant. However - ongoing funding remains a challenge. | Enrolment a challenge initially but improved over time via constant promotion/building up communication strategies/awareness. | Clear, consistent, and open communication between entities was the most-frequently reported factor contributing to successful integration. |
| Tynan, et al., 2018, AUS. | Older people in residential aged care facilities (RACF) in regional/rural settings in QLD, AUS. | To investigate the impact and experience of an integrated oral health programme using tele-dentistry and Oral Health Therapists (OHTs) in a rural setting. A mixed methods comparison study - comparing facilities with/without access to the integrated health programme. Main data sets were clinical audit data (252 audits across 9 sites) and 27 oral health quality of life surveys with eligible residents. One FGD and 8 individual interviews were completed with RACF staff. | 252 audits were completed across 9 facilities - 111 at intervention sites and 141 at non-intervention sites. Facilities that engaged with the intervention were more likely to be implementing a satisfactory oral health plan (89.2 vs 75.2%). p=0.005. Regularly replacing toothbrushes (85.6 vs68.9%) and recorded last dental visit were also significantly better in intervention sites. | Delivered directly to residents in RACFs. | Study claims that the integrated intervention helped to address inequality in access as well as improve oral health education, promotion, disease prevention and timely intervention. | Delivered directly to residents in RACFs. | Delivered directly to residents in RACFs. | Not evident | Not evident |
| Ward et al., 2022, USA. | Total of 1631 students -  164 students at seven preschool sites by Marshfield Clinic Health System (MCHS) and 1467 students at 57 school sites by Children’s dental services in USA – States that were served by the Marshfield Clinical Health System (MCHS) and Children’s Dental Services (CDS). | The School-Based Telehealth Network Grant Program (SB TNGP) was designed to expand access to, and improve  the quality of health care services in schools through telehealth. The paper describes how the two school-based teledentistry programs increased access to oral health services for children  and adolescents living in rural areas. Descriptive quantitative. | Both MCHS and CDS reported that over 99 percent of encounters were successfully completed using telehealth  technology. Both grantees reported that 99.4 percent of students received an oral health evaluation/screening, primarily  through a dental hygienist traveling to the school site. | Costs not specifically reported and unclear and if costs were associated to the users. Discussion indicates that telehealth offers savings due to reduced travel costs and lower salaries of dental professions. | The selection of HS specific programs was based on MCHS’s internal assessment of community needs. | Increased access reported with increased oral health services provided such as fluoride varnish application. | The CDS groups appear to deliver more encounters and interventions than MCHS sites.  Needs were high percentage of low-income Hispanic/Latinx families. | Sex, race, ethnicity and age group reported. | Not evident |
| Weinstein et al., 2014, USA. | 400 English-speaking women and their live-born children living in four rural communities in Oregon State USA | To describe the design, training, and methods of ongoing fidelity monitoring in the Community-Based Intergenerational Oral Health Intervention Study “Baby Smiles”. Randomized clinical trial | The frequency of MI occurrences in the delivery of the HE condition was very low compared to the frequencies in the MI condition. The competence of MI delivery did not differ among interventionists. All counsellors had much higher frequencies of counselling behaviours such as in the MI than in the HE conditions. | Not evident | Unclear | The intervention was delivered in a Women, Infants, and Children Center (WIC) or a public health department. | The goal of the HE intervention was to improve oral health-related behaviour also, but not through a patient-centred dialogue. In the HE conditions, counsellors played videos, stopped the videos at prescribed points to ask, “Do you have any questions?” and provided the participant with print materials from the National Maternal Child Oral Health Resource Center at Georgetown University (http://www.mchoralhealth.org). | There were no differences between the ratings of HE and MI participants overall or by individual counsellor (p > .05). Average feedback varied by counsellor (p = .001), and although the differences were statistically significant for counsellors 1 vs. 2 (.4) and counsellors 1 vs. 3 (.5), the magnitude of the difference was clinically negligible. | Participants in both intervention groups received printed handouts about how to use their dental care insurance coverage, what to expect at a dental visit and other written recommendations developed for the study. |
| Wetterhall et al., 2011, USA. | Key stakeholders (e.g., village leaders, school personnel, clinic staff) and direct observation to assess community based preventive efforts and overall community context in Alaska, USA. Caregivers of 233 children aged 6 to 17 years who had been treated in the previous 12 months by a DHA. | To evaluate the DHAT program in Alaska related to the quality of care, patient satisfaction, and implementation of preventive practices. Mixed methods study. | Caregivers’ rating [on a scale of 0 (worst) to 10 (best)] for dental care in the previous 12 months for children who visited DHATs was 8.24 (SD = 2.16); for children who were also treated by another dental provider, it was 8.05 (SD = 2.35). The majority of children’s caregivers had positive responses about DHATs’ communication skills and chairside manner | Financial backing from the Rasmuson Foundation, Bethel Community Services Foundation and the endorsement of the Alaska Native Tribal Health Consortium, the tribal health associations, and several dental directors | This evaluation indicated that the use of DHATs is a promising approach to addressing the crisis of-care challenges posed by a growing shortage of dentists, particularly for sparsely populated areas. Well trained and certified non dentist providers, who live in the villages where they practice full time, augment dental services in Alaska. | Increased access to oral care among rural people. | Improved the access to emergency care. Changing individual attitudes to value preventive dental care. The DHAT model minimizes impediments such as travel requirements and weather and makes access to emergency and regularly scheduled restorative and preventive care a viable option. | The rapport between DHATs and patients made the patients less fearful of the procedures. Results indicated that patients satisfied with the care they received. | Mentions clear consistent and open communication. |
| Wooley, 2016, AUS. | Children of the 5/ 6-year-old age group, 0–4 year- olds Nganampa Health Council Dental Program, AUS. | To assess the Nganampa Health Council Dental Program. Evaluation | Fissure sealant and fluoride varnish rates close to 100% in the child population. Emergency attendance rates for children are consistently less than 3% due to the comprehensive access to care of this population group, while adult emergency rates have ranged from 59– 90%. The dmft for children of the 5/ 6 year old age group has fluctuated from 3.2 in 2000, to 4.51 in 2012/ 13 (SA 2009=2.02). The dmft =0% is 16.9% on APY Lands (SA 2009 =53.3%). | Commissioned by the Aboriginal Health Organisation (AHO) of South Australia. | Internal NHC partnerships and external partnerships involved for different levels of the programme. Internal NHC partnerships with the dental program include Aboriginal Health Workers, Women’s Health/ Midwives, Child Health Program, Chronic Disease Program, Aged Care Program, and Medical Practitioner and Registered Nurse referral. External partnerships were important. | Access to care is aided by a custom- built mobile dental clinic and two dental surgeries located in the health clinics in the communities of Pukatja and Iwantja. | Key program areas have been prioritised and include Oral Health Promotion, Emergency Service, School Dental Program, Adult Dental Program, Special Needs, and Prosthodontics. | Not evident | Not evident |
| Wright, et al. 2021, USA. | Convenience sample of 200 residents aged 18 years or older in Robeson County in North Carolina, USA. | To assess the use of geofence technology to raise awareness of a dental clinic in rural North Carolina. Pre- and Postintervention Community Survey | A total of 516,073 impressions were delivered over 60 days at the cost of approximately $0.01 per impression. As a result of those impressions, 475 individuals clicked the banner to get to the website for the dental service clinic, a click-through rates of 0.09%. The total impressions delivered generated 632 sessions on the dental website, 88.9% of which were unique. | Provide tax credits to dentists to establish programs in underserved areas to provide treatment to vulnerable populations, integrating multidisciplinary collaborations within existing health systems, and raising Medicaid fees to at least the 75th percentile of dentists’ actual fees. | The School of Dental Medicine at East Carolina University provides services to patients living in rural or underserved areas across the state by providing 8 Community Service Learning Centers (CSLCs). | CSLCs are located in the rural or underserved areas across the state. | The CSLCs offer access to a full scope of dental services provided by fourth-year dental students and dental residents at a reduced cost. | A total of 475 clicks received during the awareness campaign increased awareness of the dental clinic, and the increased number of respondents who reported dental visits by the respondent or family member of the respondent suggested that the campaign had a moderate reach. | Used advanced mobile technology (mobile display via geofencing) to disseminate information about the clinic. Although there was an increase in awareness of the clinic, there was no evidence of an increase in the utilization of dental services. While awareness is a necessary first step in oral health care utilization, having dental providers located in the immediate vicinity does not guarantee individuals will receive the oral health care they need |
| Zaror, et al., 2020, CHILE. | 275 of 2- and 3-year-old children from a low socioeconomic background, living in rural areas in the Chilean Regions of La Araucanía, Los Ríos and Los Lagos CHILE. | To evaluate the incremental cost-effectiveness of the community-wide application of fluoride varnish in preschool children in rural areas with no access to fluoridated water from a public payer perspective and thus provide evidence to support or refocus this public health policy. Two-year follow-up triple blind randomized control trial. Rural areas in the Chilean Regions of La Araucanía, Los Ríos and Los Lagos in Chile. | Incidence of ECC and DMFT were 45% and 1.6 for the varnish group and they were 55.6% and 2.1 for the placebo group with a two-year follow-up. The only difference in cost between one protocol and the other is CLP 9320 (USD 13.58), which corresponds to the 4 fluoride varnish applications in the total 24-month follow-up. The sensitivity analysis showed that the increase in caries was the variable which most influenced the ICER. | Resources from the Chilean Ministry of Health, but administered by the municipalities that hire the human resources and purchase the supplies | Not evident | The intervention was conducted in public rural preschools in areas without access to fluoridated water. This population is highly vulnerable due to its characteristics of age, rurality and lack of access to fluoridated drinking water. | This CEA is based on real frequencies and probabilities, obtained from a clinical trial, with a suitable definition of the population, with limited resources and in areas with no access to fluoridated drinking water. | Not evident | Not evident |
