# Supplementary material for: Oral Health Interventions to Improve Access in Rural Areas of High‐Income Countries: A Mixed Methods Systematic Review
Source: Community Dent Oral Epidemiol. 2026 Feb 18;54(3):273–84. doi: 10.1111/cdoe.70058 (PMC13146141; doi:10.1111/cdoe.70058)
Supplement: Supplementary file 4 — Appendix S4: Quality appraisal of all study types. [file CDOE-54-273-s002.docx]

**Appendix 4: Quality appraisal of all study types**

**Quality Appraisal of Quantitative Studies using the MMAT Criteria**

| **Primary author, year** | **Screening questions** | |  | **Quantitative randomised controlled studies MMAT criteria** | | | | |  | **Quantitative non-randomised studies MMAT criteria** | | | | |  | **Quantitative descriptive studies MMAT criteria** | | | | | |
| --- | --- | --- | --- | --- | --- | --- | --- | --- | --- | --- | --- | --- | --- | --- | --- | --- | --- | --- | --- | --- | --- |
|  | **S1** | **S2** |  | **2.1** | **2.2** | **2.3** | **2.4** | **2.5** |  | **3.1** | **3.2** | **3.3** | **3.4** | **3.5** |  | **4.1** | **4.2** | **4.3** | **4.4** | **4.5** |  |
| Arrow 2021 | ✓ | ✓ |  | ✓ | ✓ | ✓ | X | X |  |  |  |  |  |  |  |  |  |  |  |  |  |
| Berndt 2008 | ✓ | ✓ |  |  |  |  |  |  |  | ✓ | ✓ | U | ✓ | ✓ |  |  |  |  |  |  |  |
| Biordi 2015 | ✓ | ✓ |  |  |  |  |  |  |  | ✓ | ✓ | X | ✓ | ✓ |  |  |  |  |  |  |  |
| Bradley 2010 | ✓ | ✓ |  |  |  |  |  |  |  |  |  |  |  |  |  | ✓ | X | ✓ | X | U |  |
| Carr 2008 | ✓ | ✓ |  |  |  |  |  |  |  | ✓ | U | X | ✓ | N |  |  |  |  |  |  |  |
| Chen 2007 | X | X |  |  |  |  |  |  |  |  |  |  |  |  |  |  |  |  |  |  |  |
| Croucher 2006 | ✓ | ✓ |  |  |  |  |  |  |  |  |  |  |  |  |  | U | ✓ | X | ✓ | X |  |
| Davis 2010 | X | X |  |  |  |  |  |  |  |  |  |  |  |  |  |  |  |  |  |  |  |
| Dimitropoulos 2020 | ✓ | ✓ |  |  |  |  |  |  |  |  |  |  |  |  |  | ✓ | ✓ | U | U | ✓ |  |
| Fallon 2010 | X | X |  |  |  |  |  |  |  |  |  |  |  |  |  | X |  |  |  |  |  |
| Gardner 2012 | X | X |  |  |  |  |  |  |  |  |  |  |  |  |  |  |  |  |  |  |  |
| Geiger 2019 | ✓ | ✓ |  |  |  |  |  |  |  | ✓ | U | X | X | ✓ |  |  |  |  |  |  |  |
| Harrison 2016 | ✓ | ✓ |  |  |  |  |  |  |  | ✓ | ✓ | X | U | X |  |  |  |  |  |  |  |
| Irving 2017 | ✓ | ✓ |  |  |  |  |  |  |  |  |  |  |  |  |  | ✓ | ✓ | ✓ | U | ✓ |  |
| Ju 2017 | ✓ | ✓ |  | ✓ | ✓ | ✓ | X | U |  |  |  |  |  |  |  |  |  |  |  |  |  |
| Kranz et al 2022 | ✓ | ✓ |  |  |  |  |  |  |  |  |  |  |  |  |  | ✓ | ✓ | ✓ | ✓ | ✓ |  |
| Kruger 2010 | ✓ | ✓ |  |  |  |  |  |  |  |  |  |  |  |  |  | ✓ | X | ✓ | X | ✓ |  |
| Lalloo 2021 | ✓ | ✓ |  |  |  |  |  |  |  | ✓ | ✓ | ✓ | U | ✓ |  |  |  |  |  |  |  |
| Larson 2019 | ✓ | ✓ |  |  |  |  |  |  |  |  |  |  |  |  |  | ✓ | ✓ | ✓ | ✓ | ✓ |  |
| Larrson 2023 | ✓ | ✓ |  |  |  |  |  |  |  | U | ✓ | X | X | U |  |  |  |  |  |  |  |
| Lee 2019 | ✓ | ✓ |  | ✓ | ✓ | ✓ | U | U |  |  |  |  |  |  |  |  |  |  |  |  |  |
| Lee 2017 | ✓ | ✓ |  | U | X | X | U | U |  |  |  |  |  |  |  |  |  |  |  |  |  |
| March 2023 | ✓ | X |  |  |  |  |  |  |  |  |  |  |  |  |  |  |  |  |  |  |  |
| Masoe 2014 | ✓ | ✓ |  |  |  |  |  |  |  |  |  |  |  |  |  | ✓ | ✓ | ✓ | ✓ | ✓ |  |
| Mathu-Muju 2016 | ✓ | ✓ |  |  |  |  |  |  |  |  |  |  |  |  |  | ✓ | ✓ | ✓ | ✓ | ✓ |  |
| Mathu-Muju 2018 | ✓ | ✓ |  |  |  |  |  |  |  |  |  |  |  |  |  | ✓ | ✓ | ✓ | ✓ | ✓ |  |
| Maurizio 2003 | X | X |  |  |  |  |  |  |  |  |  |  |  |  |  |  |  |  |  |  |  |
| Mays 2018 | ✓ | ✓ |  |  |  |  |  |  |  |  |  |  |  |  |  | ✓ | ✓ | ✓ | ✓ | ✓ |  |
| Kaakko et al., 2002  Meihubers 2013 | ✓  X | ✓  X |  | U | U | Y | U | U |  |  |  |  |  |  |  |  |  |  |  |  |  |
| Milgrom 2017 | ✓ | X |  |  |  |  |  |  |  |  |  |  |  |  |  |  |  |  |  |  |  |
| Ng 2023 | ✓ | ✓ |  |  |  |  |  |  |  |  |  |  |  |  |  | ✓ | U | ✓ | N/A | ✓ |  |
| Nycz 2020 | X | X |  |  |  |  |  |  |  |  |  |  |  |  |  |  |  |  |  |  |  |
| Pacza 2001 | X | X |  |  |  |  |  |  |  |  |  |  |  |  |  |  |  |  |  |  |  |
| Patel 2015 | ✓ | ✓ |  |  |  |  |  |  |  |  |  |  |  |  |  | ✓ | ✓ | ✓ | ✓ | ✓ |  |
| Ramos-Gomez 2024 | ✓ | ✓ |  |  |  |  |  |  |  |  |  |  |  |  |  | U | ✓ | ✓ | NA | ✓ |  |
| Ragade et al. 2022 | ✓ | ✓ |  |  |  |  |  |  |  | ✓ | ✓ | ✓ | ✓ | ✓ |  |  |  |  |  |  |  |
| Roberts 2016 | X | X |  |  |  |  |  |  |  |  |  |  |  |  |  |  |  |  |  |  |  |
| Roberts-Thompson 2010 | ✓ | ✓ |  | ✓ | ✓ | ✓ | X | X |  |  |  |  |  |  |  |  |  |  |  |  |  |
| Rowland 2016 | ✓ | ✓ |  |  |  |  |  |  |  |  |  |  |  |  |  | ✓ | ✓ | ✓ | ✓ | ✓ |  |
| Ruff and Niederman 2024 | ✓ | ✓ |  | ✓ | ✓ | X | X | X |  |  |  |  |  |  |  |  |  |  |  |  |  |
| Schroeder 2021 | ✓ | ✓ |  |  |  |  |  |  |  |  |  |  |  |  |  | X | X | ✓ | X | ✓ |  |
| Shah 2024 | ✓ | ✓ |  |  |  |  |  |  |  |  |  |  |  |  |  | ✓ | ✓ | ✓ | NA | ✓ |  |
| Shimpi 2024 | ✓ | ✓ |  |  |  |  |  |  |  |  |  |  |  |  |  | ✓ | ✓ | ✓ | NA | ✓ |  |
| Siegal 2010 | ✓ | ✓ |  |  |  |  |  |  |  |  |  |  |  |  |  | ✓ | ✓ | ✓ | ✓ | ✓ |  |
| Skelton 2009 | ✓ | ✓ |  |  |  |  |  |  |  |  |  |  |  |  |  | ✓ | ✓ | ✓ | ✓ | ✓ |  |
| Skinner 2020 | ✓ | ✓ |  |  |  |  |  |  |  |  |  |  |  |  |  | ✓ | ✓ | ✓ | X | ✓ |  |
| Stewart 2022 | X | X |  |  |  |  |  |  |  |  |  |  |  |  |  |  |  |  |  |  |  |
| Surdu 2020 | ✓ | ✓ |  |  |  |  |  |  |  |  |  |  |  |  |  | ✓ | U | ✓ | U | ✓ |  |
| Ward 2022 | X | ✓ |  |  |  |  |  |  |  |  |  |  |  |  |  |  |  |  |  |  |  |
| Weinstein 2014 | ✓ | ✓ |  | ✓ | ✓ | ✓ | X | U |  |  |  |  |  |  |  |  |  |  |  |  |  |
| Wetterhall 2011 | X | X |  |  |  |  |  |  |  |  |  |  |  |  |  |  |  |  |  |  |  |
| Wooley 2016 | ✓ | X |  |  |  |  |  |  |  |  |  |  |  |  |  |  |  |  |  |  |  |
| Wright 2021 | ✓ | ✓ |  |  |  |  |  |  |  |  |  |  |  |  |  | ✓ | X | ✓ | X | ✓ |  |
| Zaror 2020 | ✓ | ✓ |  | U | U | U | U | U |  |  |  |  |  |  |  |  |  |  |  |  |  |

**Abbreviations**: ✓=Yes, X =No, U=Unclear, N/A=Not applicable

MMAT Quality Appraisal Criteria:

**Screening questions:**

S1. Are there clear research questions?

S2. Do the collected data allow to address the research questions?

**Quantitative randomised controlled trials**

2.1. Is randomization appropriately performed?

2.2. Are the groups comparable at baseline?

2.3. Are there complete outcome data?

2.4. Are outcome assessors blinded to the intervention provided?

2.5 Did the participants adhere to the assigned intervention?

**Quantitative non-randomised**

3.1. Are the participants representative of the target population?

3.2. Are measurements appropriate regarding both the outcome and intervention (or exposure)?

3.3. Are there complete outcome data?

3.4. Are the confounders accounted for in the design and analysis?

3.5. During the study period, is the intervention administered (or exposure occurred) as intended?

**Quantitative descriptive**

4.1. Is the sampling strategy relevant to address the research question?

4.2. Is the sample representative of the target population?

4.3. Are the measurements appropriate?

4.4. Is the risk of nonresponse bias low?

4.5. Is the statistical analysis appropriate to answer the research question?

**Quality appraisal of Qualitative Studies using the MMAT**

| **Primary author, year** | **Screening questions** | |  | **MMAT criteria for Qualitative studies** | | | | |
| --- | --- | --- | --- | --- | --- | --- | --- | --- |
|  |  | |  |  | | | | |
|  | S1 | S2 |  | 1.1 | 1.2 | 1.3 | 1.4 | 1.5 |
| Bright & Lichtman 2024  Castillo et al., 2023  Chi 2019 | ✓  ✓  ✓ | ✓  X  ✓ |  | ✓  ✓ | ✓  ✓ | ✓  ✓ | ✓  ✓ | ✓  ✓ |
| Delinger 2014 | ✓ | ✓ |  | ✓ | ✓ | ✓ | ✓ | ✓ |
| Mangoyana 2023 | ✓ | ✓ |  | ✓ | ✓ | ✓ | ✓ | ✓ |
| Patel 2021 | ✓ | ✓ |  | ✓ | ✓ | ✓ | ✓ | ✓ |
| Pawlowski 2022 | ✓ | ✓ |  | ✓ | ✓ | ✓ | ✓ | ✓ |
| Piggott 2021 | ✓ | ✓ |  | ✓ | ✓ | ✓ | ✓ | ✓ |
| Rajabiun 2011 | ✓ | ✓ |  | ✓ | ✓ | ✓ | ✓ | ✓ |
| Senturia 2018 | ✓ | ✓ |  | ✓ | ✓ | ✓ | ✓ | ✓ |

**Abbreviations**: ✓=Yes, X= No.

MMAT Quality Appraisal Criteria [94]:

**Screening questions:**

S1. Are there clear research questions?

S2. Do the collected data allow to address the research questions?

**Qualitative**

1.1 Is the qualitative approach appropriate to answer the research question?

1.2 Are the qualitative data collection methods adequate to address the research question?

1.3 Are the findings adequately derived from the data?

1.4 Is the interpretation of results sufficiently substantiated by data?

1.5 Is there coherence between qualitative data sources, collection, analysis and interpretation?

**Quality Appraisal of Mixed Method Studies using the MMAT Criteria**

| **Primary author, year** | **Screening questions** | | | **MMAT criteria for Mixed Methods studies** | | | | |
| --- | --- | --- | --- | --- | --- | --- | --- | --- |
|  |  | | |  | | | | |
|  | | **1.1** | **1.2** | **5.1** | **5.2** | **5.3** | **5.4** | **5.5** |
| Bryant 2016 | | ✓ | ✓ | ✓ | ✓ | U | X | ✓ |
| Gaskin 2018 | | ✓ | ✓ | ✓ | ✓ | ✓ | ✓ | U |
| Gnaedinger 2018 | | ✓ | ✓ | U | ✓ | ✓ | X | ✓ |
| Javed et al., 2024 | | ✓ | ✓ | ✓ | ✓ | X | X | U |
| Jones 2012 | | X | X |  |  |  |  |  |
| Trudnak 2019 | | ✓ | ✓ | ✓ | ✓ | ✓ | X | ✓ |
| Tynan 2018 | | ✓ | ✓ | ✓ | ✓ | ✓ | X | ✓ |
| Spetz 2019 | | ✓ | ✓ | ✓ | ✓ | ✓ | X | ✓ |

**Abbreviations**: ✓=Yes, X =No, U=Unclear, N/A=Not applicable

MMAT Quality Appraisal Criteria:

**Screening questions:**

S1. Are there clear research questions?

S2. Do the collected data allow to address the research questions?

**Mixed Methods**

5.1. Is there an adequate rationale for using a mixed methods design to address the research question?

5.2. Are the different components of the study effectively integrated to answer the research question?

5.3. Are the outputs of the integration of qualitative and quantitative components adequately interpreted?

5.4. Are divergences and inconsistencies between quantitative and qualitative results adequately addressed?

5.5. Do the different components of the study adhere to the quality criteria of each tradition of the methods involved?
